# Functional validation of a human *GLUD2* variant in a murine model of Parkinson’s disease

**DOI:** 10.1038/s41419-020-03043-2

**Published:** 2020-10-22

**Authors:** Wenlong Zhang, Junwei Gong, Liuyan Ding, Zhiling Zhang, Xiaowen Pan, Xiang Chen, Wenyuan Guo, Xiaokang Zhang, Xinling Yang, Guoyou Peng, Yuwan Lin, Feng Gao, Yuanquan Li, Xiaoqin Zhu, Aiguo Xuan, Shu Wang, Xiangdong Sun, Yunlong Zhang, Pingyi Xu

**Affiliations:** 1 Department of Neurology, The First Affiliated Hospital of Guangzhou Medical University, 510120 Guangzhou, China; 2 Key Laboratory of Neurological Function and Health, School of Basic Medical Sciences, Guangzhou Medical University, 511436 Guangzhou, China; 3 The First Affiliated Hospital of Gannan Medical University, 341000 Ganzhou, China; 4 Department of Neurology, The Second Affiliated Hospital of Xinjiang Medical University, 830011 Urumqi, China; 5 Department of Physiology, School of Basic Medical Sciences, Guangzhou Medical University, 511436 Guangzhou, China

**Keywords:** Genetics of the nervous system, Molecular neuroscience, Parkinson's disease

## Abstract

Parkinson’s disease (PD) is a common neurodegenerative disease characterized by Lewy body formation and progressive dopaminergic neuron death in the substantia nigra (SN). Genetic susceptibility is a strong risk factor for PD. Previously, a rare gain-of-function variant of *GLUD2* glutamate dehydrogenase (T1492G) was reported to be associated with early onset in male PD patients; however, the function and underlying mechanism of this variant remains elusive. In the present study, we generated adeno-associated virus expressing *GLUD2* and its mutant under the control of the glial fibrillary acidic protein promotor and injected the virus into the SN pars compacta of either untreated mice or 1-methyl-4-phenyl-1,2,3,6-tetrahydropyridine (MPTP)-induced PD model mice. Our results demonstrate that *GLUD2* mutation in MPTP-induced PD mice exacerbates movement deficits and nigral dopaminergic neuron death and reduces glutamate transporters expression and function. Using GC-Q-TOF/MS-based metabolomics, we determined that *GLUD2* mutation damages mitochondrial function by decreasing succinate dehydrogenase activity to impede the tricarboxylic acid cycle in the SN of MPTP-induced PD mice. Accordingly, *GLUD2* mutant mice had reduced energy metabolism and increased apoptosis, possibly due to downregulation of brain-derived neurotrophic factor/nuclear factor E2-related factor 2 signaling in in vitro and in vivo PD models. Collectively, our findings verify the function of *GLUD2* in PD and unravel a mechanism by which a genetic variant in human *GLUD2* may contribute to disease onset.

## Introduction

Parkinson’s disease (PD) is a common chronic neurodegenerative disease that affects 1.8% of the world’s population over the age of 65 and is associated with genetic and environmental risk factors^1^. The clinical characteristics of PD include movement disorder (e.g., rigidity, tremors, bradykinesia) and non-motor symptoms (e.g., depression, constipation, genitourinary problems, sleep disorders)^2^. Additional pathological hallmarks of PD include age-dependent loss of dopaminergic (DA) neurons in the substantia nigra (SN) and Lewy body formation caused by aggregated α-synuclein. PD pathogenesis is associated with mitochondrial dysfunction, protein misfolding, oxidative stress, glutamate dysfunction, and genetic susceptibility^3–5^. Furthermore, current evidence supports the association of several genes, including *SNCA*, *LRRK2*, *VPS35*, *PRKN*, *PINK1*, *GBA*, and *DJ-1*, with PD^6–9^; however, additional genes that harbor rare mutations in PD still need functional validation.

In the central nervous system, normal glutamate metabolism plays an important role in signal transmission and brain function^10^. Excitatory amino acid transporters (EAATs), located on the membranes of astrocytes, take up and recycle a large proportion of the synaptic glutamate, which is converted to glutamine or used in mitochondrial metabolism^11^. Intracellularly, glutamate dehydrogenase (GDH), which resides in mitochondria, catalyzes the reversible reaction of glutamate and α-ketoglutaric acid and participates in a variety of cellular physiological processes, including the tricarboxylic acid (TCA) cycle, ammonia metabolism regulation, energy generation and signal transduction with coenzymes NAD(H) and NADP(H)^12,13^. Most mammals express GDH1 (encoded by *GLUD1*), while humans and other primates also possess the highly homologous GDH2 (encoded by *GLUD2*), which differs from GDH1 only in 15 out of 505 amino acid residues^14,15^. Generally, hGDH1 is widely expressed in human tissue, while hGDH2 expression is localized to Sertoli cells in the testis, astrocytes in the brain, and epithelial cells in the kidney^16,17^. Specifically, in the mammalian brain, GDH1 is expressed in both neurons and astrocytes^18^, while hGDH2 is expressed in astrocytes rather than neurons^16,19^. Also, hGDH2 expression is suggested to affect glutamate and TCA cycle metabolism in astrocytes^20^, and abnormalities in glutamate metabolism have been reported in patients with neurodegenerative disease, such as PD^21,22^, with deficiency of GDH activity observed in many PD patients^23^. In 2010, Plaitakis et al. reported that a rare T1492G variant of the X-linked *GLUD2* gene hastens the onset of PD in male patients^24^. They demonstrated that this variant may enhance glutamate oxidative dehydrogenation; however, the precise underlying mechanisms remain elusive.

In this study, to evaluate the effect of T1492G mutation on *GLUD2* function, we generated adeno-associated viruses (AAVs) that express *GLUD2* or the corresponding mutant under the regulation of the glial fibrillary acidic protein (GFAP) promotor and injected the viruses in the SN pars compacta (SNpc) of normal and 1-methyl-4-phenyl-1,2,3,6-tetrahydropyridine (MPTP)-induced PD model mice. Here, we report that *GLUD2* mutation aggravates motor deficits and nigral DA neuron death in MPTP-treated mice. We also reveal that the underlying mechanism may be explained by *GLUD2* mutant-associated reduction in glutamate transporters expression, which worsens the defect induced by MPTP on the mitochondrial complex I, affecting complex II/succinate dehydrogenase (SDH) activity and increasing apoptosis in AAV-GLUD2 T1492G mice. These effects of *GLUD2* mutation may be caused by downregulation of brain-derived neurotrophic factor (BDNF)/nuclear factor E2-related factor 2 (Nrf2) signaling, which was demonstrated both in MPTP-treated mice and in 1-methyl-4-phenylpyridinium-iodide (MPP^+^)-treated U251 cells. Thus, our findings provide functional evidence that the T1492G variant of *GLUD2* may modify disease onset in a male PD model.

## Results

### *GLUD2* mutation aggravates movement deficits in MPTP-treated mice

**Fig. 1:**
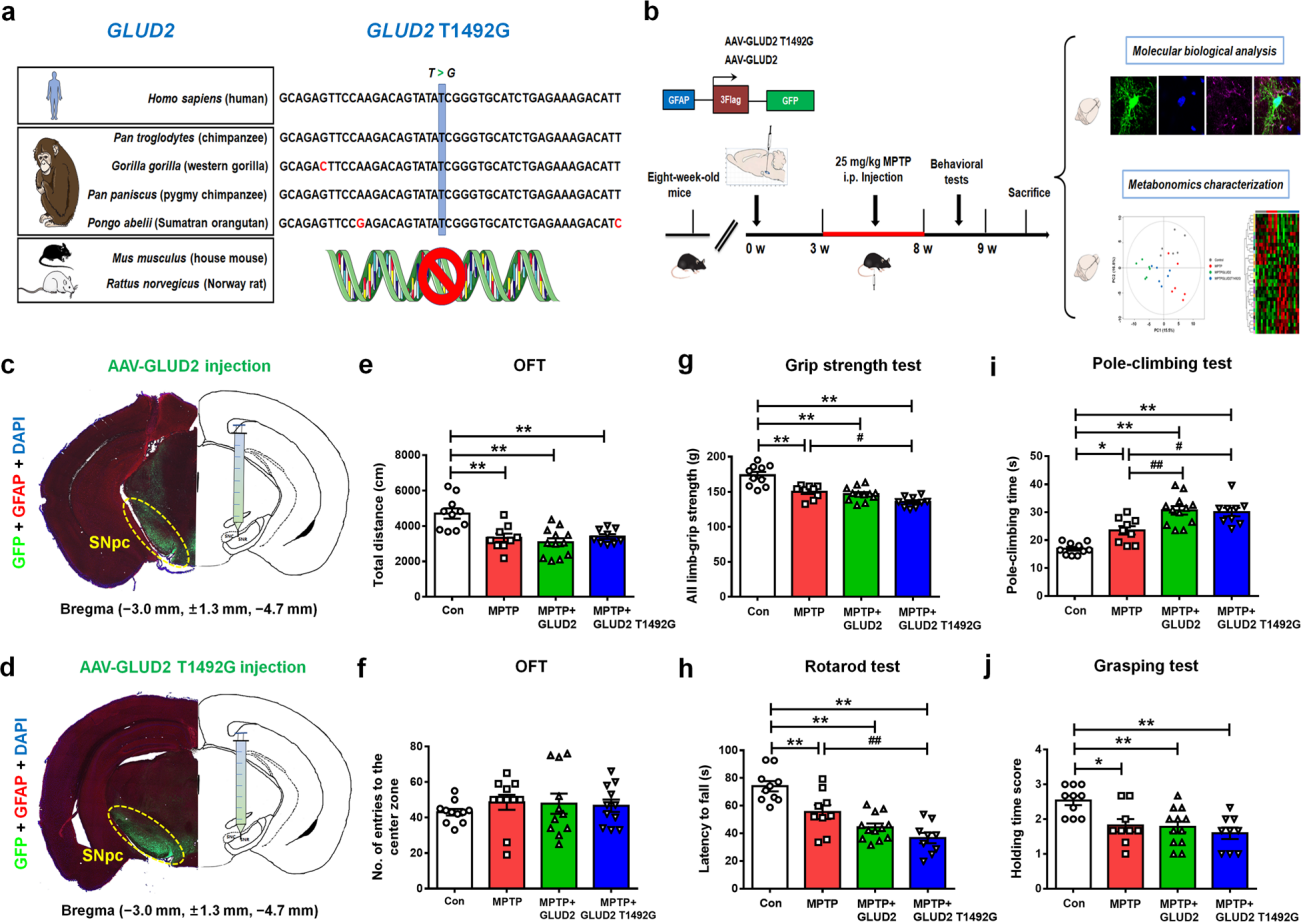
*GLUD2* mutation aggravates the movement disorder in MPTP-treated mice. **a** The *GLUD2* gene sequences across humans and other primates are shown. Blue coloring highlights the high homology of the T1492 site. **b** The experimental design of this study is shown. AAV-GLUD2, AAV-GLUD2 T1492G, or AAV-GFP was stereotaxically injected into the SNpc, and 3 weeks later, mice were intraperitoneally administered saline or MPTP for 5 weeks. One day after the last MPTP/saline injection, behavioral tests were performed, after which time the mice were sacrificed. (**c**, **d**) The expression of AAV-GLUD2 and AAV-GLUD2 T1492G in the SNpc was verified by immunostaining with GFP and GFAP antibodies. **e**, **f** Total distances traveled and numbers of entries to the center zone in the open-field are shown for AAV-GFP, MPTP + AAV-GFP, MPTP + AAV-GLUD2, and MPTP + AAV-GLUD2 T1492G groups. **g** The grip strength test was used to examine the limb grip strength of mice. **h** The rotarod test was used to examine the motor coordination of mice. **i** The pole-climbing test was used to examine the bradykinesia of mice. **j** The grasping test was used to further examine the grip strength of mice. n = 10, 9, 12, and 9 for AAV-GFP, MPTP + AAV-GFP, MPTP + AAV-GLUD2, and MPTP + AAV-GLUD2 T1492G groups, respectively. Results are expressed as the mean ± SEM. ^**^p < 0.01, ^*^p < 0.05 vs. AAV-GFP group. ^##^*p* < 0.01, ^#^*p* < 0.05 vs. MPTP + AAV-GFP group. Statistical significance was determined by one-way ANOVAs and Tukey tests for *post-hoc* comparisons.

The T1492 nucleotide in the *GLUD2* gene, which is possessed by humans and other primates but not by rodents^15^, is highly conserved across these species (Fig. 1a). To determine whether the *GLUD2* variant (T1492G) affects PD pathogenesis, we generated AAV constructs for expression of *GLUD2* or the *GLUD2* mutant (AAV-GLUD2 or AAV-GLUD2 T1492G). Because hGDH2 expression is restricted to astrocytes, we designed the constructs to express these genes under the control of a GFAP promoter. As a control, we used an AAV GFAP promoter construct expressing GFP (AAV-GFP). We then delivered these viruses into the bilateral SNpc. Three weeks later, we administered MPTP or saline for another 5 weeks (Fig. 1b). We verified the expression of AAV-GLUD2 and AAV-GLUD2 T1492G virus in the SNpc region by staining the corresponding Flag GFP-tagged proteins with GFAP (Fig. 1c, d). To evaluate the effects of *GLUD2* mutation in the MPTP-induced PD model, we performed behavioral tests. The total distance traveled was decreased in each of the MPTP-treated groups as compared with the AAV-GFP control group (Fig. 1e), thus verifying the successful application of the MPTP-induced PD model. While these four groups showed no significant changes regarding entries into the center zone of the open field (Fig. 1f), the behavioral indicators, as evaluated by the grip strength, rotarod, pole-climbing and grasping tests, each revealed impaired motor function for the three groups of MPTP-treated mice as compared with untreated AAV-GFP group (Fig. 1g–j); however, the effect of the grip strength and rotarod tests was more dramatic for the MPTP + AAV-GLUD2 T1492G group than the MPTP + AAV-GFP group (Fig. 1g, h), and the effect of the pole-climbing test was more dramatic for both the MPTP + AAV-GLUD2 and MPTP + AAV-GLUD2 T1492G groups than for the MPTP + AAV-GFP group (Fig. 1i). These results suggest that the *GLUD2* T1492G mutant aggravates MPTP-induced motor damage.

To determine whether expression of *GLUD2* T1492G also affects the motor function of naive mice in the absence of MPTP treatment, we delivered the adenoviruses into the SNpc of naive mice. AAV-GLUD2 and its mutant virus showed no obvious effects on the total distance traveled, duration of time spent in the center zone or the number of entries to the center zone in the open-field test (Supplementary Fig. 1a–c). Furthermore, AAV-GLUD2 and its mutant virus did not affect the pole-climbing, rotarod or grasping test results (Supplementary Fig. 1d–f). These results suggest that while the expression of the *GLUD2* mutant exacerbates the motor damage induced by MPTP in mice, its expression has no apparent effect in the absence of MPTP treatment.

### *GLUD2* mutation exacerbates the reduction of nigral DA neurons in MPTP-treated mice

**Fig. 2:**
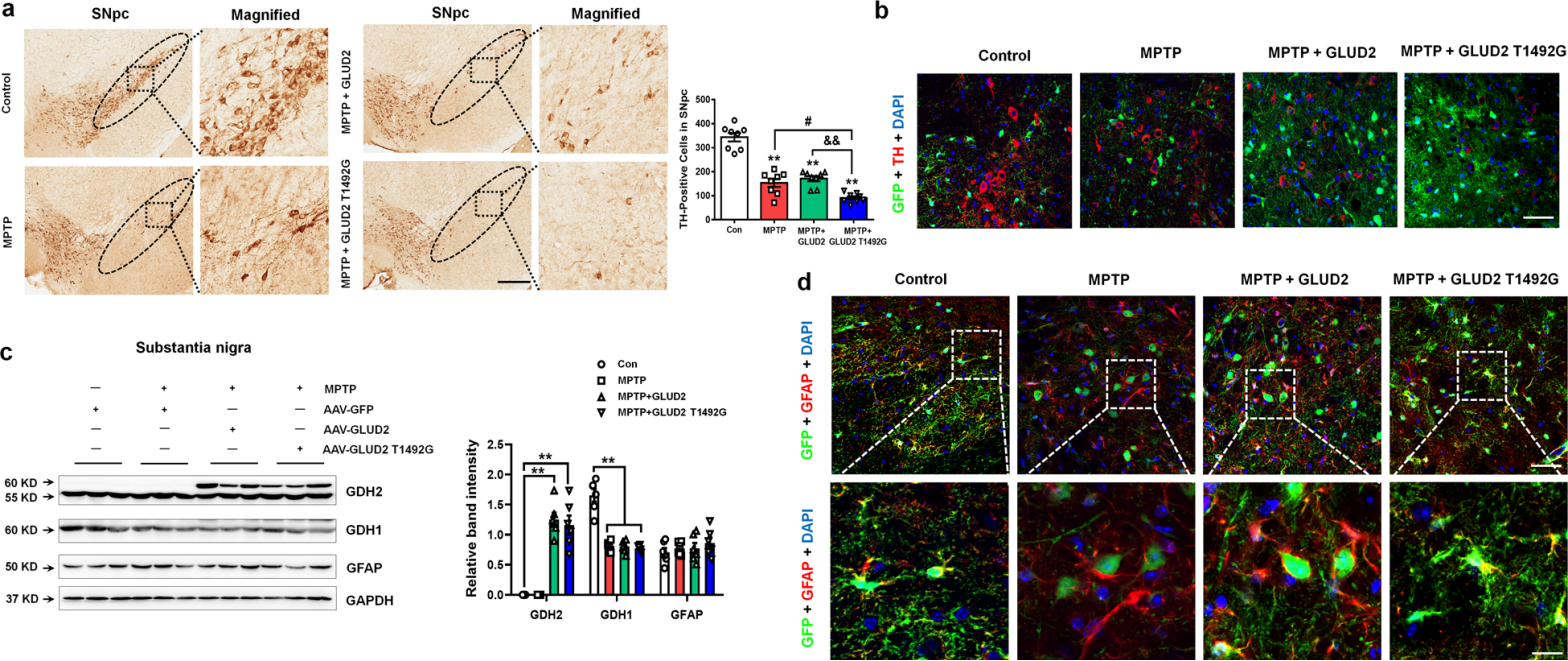
*GLUD2* mutation exacerbates nigral cell death in MPTP-treated mice. **a** Immunohistochemical staining of TH-positive cells in the SNpc in AAV-GFP, MPTP + AAV-GFP, MPTP + AAV-GLUD2, and MPTP + AAV-GLUD2 T1492G groups (scale bars, 100 µm). The ellipses in the middle column of (**a**) show the boundaries of the SNpc, and the middle-column boxes denote the areas that are expanded in the right-hand columns. *n* = 8. **b** Immunofluorescent staining of GFP and TH in the SNpc of AAV-GFP, MPTP + AAV-GFP, MPTP + AAV-GLUD2, and MPTP + AAV-GLUD2 T1492G groups (scale bars, 40 µm). **c** Expression levels of GDH2, GDH1, and GFAP in the SN of AAV-GFP, MPTP + AAV-GFP, MPTP + AAV-GLUD2, and MPTP + AAV-GLUD2 T1492G groups were determined by western blotting. The bottom band (~55 KD) in the GDH2 blot is non-specific. *n* = 6. **d** Immunofluorescent staining of GFP and GFAP in the SNpc of AAV-GFP, MPTP + AAV-GFP, MPTP + AAV-GLUD2, and MPTP + AAV-GLUD2 T1492G groups (scale bars, upper, 40 µm; lower, 10 µm). Results are expressed as the mean ± SEM. ^**^p < 0.01 vs. AAV-GFP group or untreated U251 cells. ^#^*p* < 0.05 vs. MPTP + AAV-GFP or MPP^+^ group. ^&&^*p* < 0.01 vs. MPP^+^ + GLUD2 group. Statistical significance was determined by one-way ANOVAs and Tukey tests for *post-hoc* comparisons.

Next, we examined the effects of *GLUD2* or its mutant on pathological changes in DA neurons. The expression of the DA neuron marker tyrosine hydroxylase (TH) in the nigra was significantly decreased in the three groups of MPTP-treated mice as compared with the untreated AAV-GFP group, and the reduction of TH was more dramatic for the MPTP + AAV-GLUD2 T1492G group than the MPTP + AAV-GFP group, as assessed by immunohistochemical staining (Fig. 2a) and immunofluorescence assay (Fig. 2b). We also confirmed overexpression of AAV-GLUD2 and its mutant in the SNpc by examining levels of the GDH2 protein that are encoded by the *GLUD2* gene (Fig. 2c). Furthermore, the western blotting assay revealed that GDH1 expression was decreased in each of the MPTP-treated groups as compared with the AAV-GFP control group, though no effects were observed on endogenous GFAP expression (Fig. 2c). Consistently, we found detectable expression of *GLUD2* and its mutant in astrocytes in the SNpc (Fig. 2d). The effects of MPTP in reducing TH expression in DA neurons were also observed in the striatum (Supplementary Fig. 2a–c). Notably, expression of the dopamine transporter DAT was significantly decreased in the MPTP + AAV-GLUD2 T1492G group as compared with the AAV-GFP or MPTP + AAV-GFP group (Supplementary Fig. 2b). Furthermore, no obvious differences in the *GLUD2*-expressing mice were observed in the expression of nigral GDH1, nigrostriatal GFAP, TH or DAT in mice that were not treated with MPTP (Supplementary Fig. 3a, b).

### *GLUD2* mutation reduces astroglial glutamate transporters expression in vivo and in vitro

**Fig. 3:**
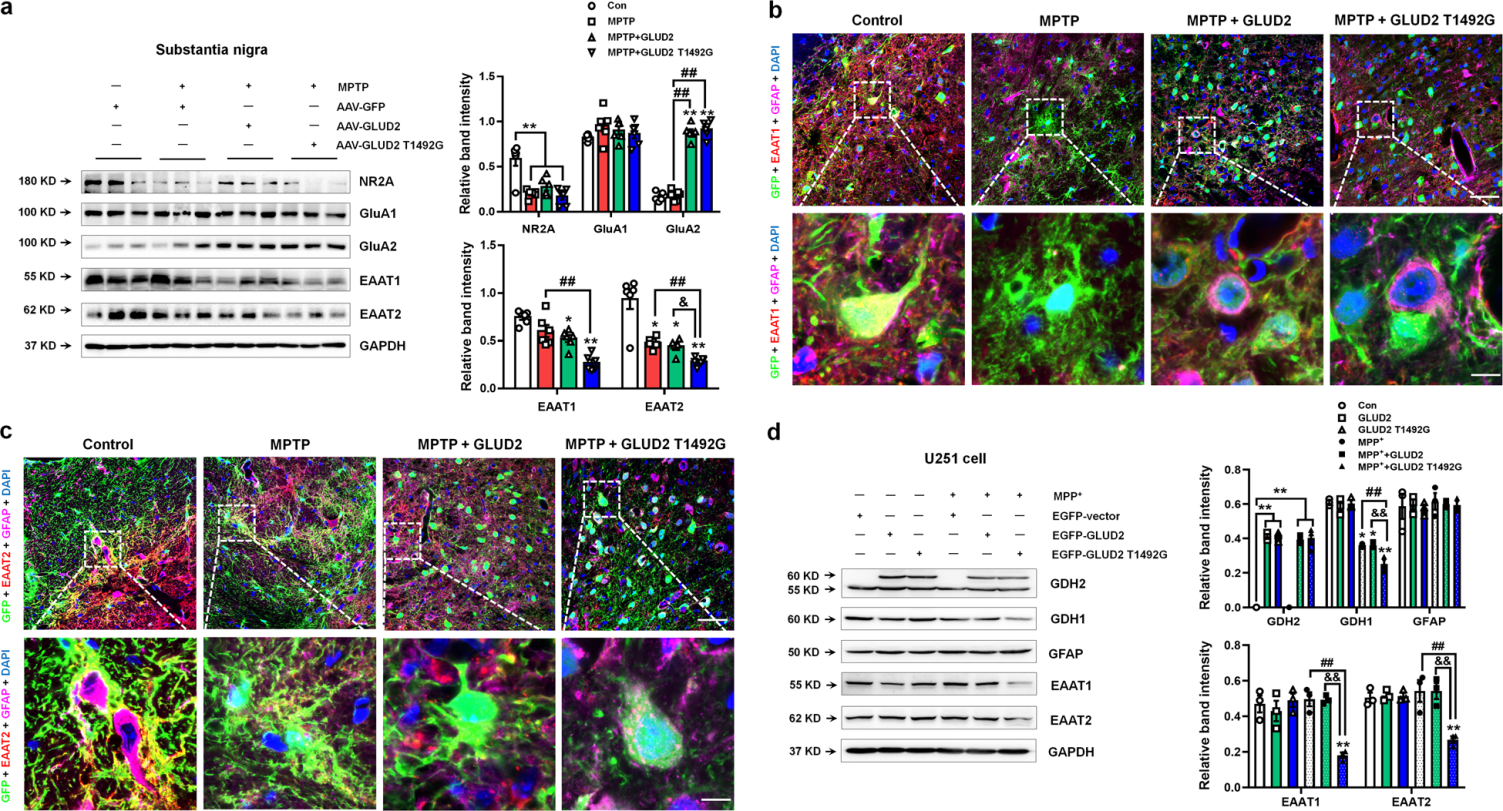
*GLUD2* mutation decreases glutamate transporter expression in MPTP-treated mice. **a** Expression levels of NR2A, GluA1, GluA2, EAAT1, and EAAT2 in the SN of AAV-GFP, MPTP + AAV-GFP, MPTP + AAV-GLUD2, and MPTP + AAV-GLUD2 T1492G groups of mice were determined by western blotting. *n* = 6. **b**, **c** Immunofluorescent staining of GFP, GFAP, and EAAT1 or EAAT2 in the SNpc of AAV-GFP, MPTP + AAV-GFP, MPTP + AAV-GLUD2, and MPTP + AAV-GLUD2 T1492G groups (scale bars: upper, 40 µm; lower, 8 µm). **d** Effect of *GLUD2* or its mutant on the expression of GDH2, GDH1, GFAP, EAAT1 and EAAT2 in MPP^+^-treated U251 cells was determined by western blotting. The bottom band (~55 KD) in the GDH2 blot is non-specific. *n* = 3. Results are expressed as the mean ± SEM. ^**^p < 0.01, ^*^p < 0.05 vs. AAV-GFP group or untreated U251 cells. ^##^*p* < 0.01 vs. MPTP + AAV-GFP or MPP^+^ group. ^&&^*p* < 0.01, ^&^*p* < 0.05 vs. MPP^+^ + GLUD2 group. Statistical significance was determined by one-way ANOVAs and Tukey tests for post-hoc comparisons.

Because the GDH2 protein participates in glutamate metabolism, we also examined glutamate receptor/transporter expression in *GLUD2*-expressing mice. The results demonstrate that MPTP treatment decreased NR2A expression and increased GluA2 expression (Fig. 3a). Furthermore, the *GLUD2* T1492G mutant significantly decreased the expression of astroglial glutamate transporters EAAT1 (also called glutamate aspartate transporter, GLAST) and EAAT2 (also called glutamate transporter-1, GLT-1) in MPTP-treated mice (Fig. 3a). Because the EAATs are mainly responsible for excessive glutamate uptake in the synaptic cleft^25^, these results suggest a mechanism by which *GLUD2* mutation may reduce glutamate metabolism. The effects of *GLUD2* mutation on glutamate transporters expression were verified by triple staining of GFP, GFAP and EAAT1 or EAAT2 in the SNpc (Fig. 3b, c). In addition, we also found that *GLUD2* mutation further decreased glutamate uptake in the synaptosomes of SN in MPTP mice (Supplementary Fig. 4a).

We next examined the effects of *GLUD2* mutation on glutamate transporters in the glioma cell line U251, which expresses endogenous EAAT transporters^26^. Toward this purpose, we generated EGFP-tagged plasmids to overexpress *GLUD2* and its mutant, with an EGFP-tagged vector as control. GDH1 expression was decreased in MPP^+^, MPP^+^ + GLUD2 and MPP^+^ + GLUD2 T1492G groups (Fig. 3d), which is consistent with the in vivo results. Furthermore, *GLUD2* T1492G mutation decreased EAAT1 and EAAT2 expression and glutamate uptake in MPP^+^-treated U251 cells as compared with the control and MPP^+^ groups (Fig. 3d and Supplementary Fig. 4b). *GLUD2* and its mutant showed no obvious effects on GFAP expression, which is also consistent with the in vivo results and provides a mechanism that could explain the *GLUD2* T1492G-dependent decrease in glutamate metabolism and uptake.

### *GLUD2* mutation damages mitochondrial metabolism by targeting SDH in MPTP-treated mice

**Fig. 4:**
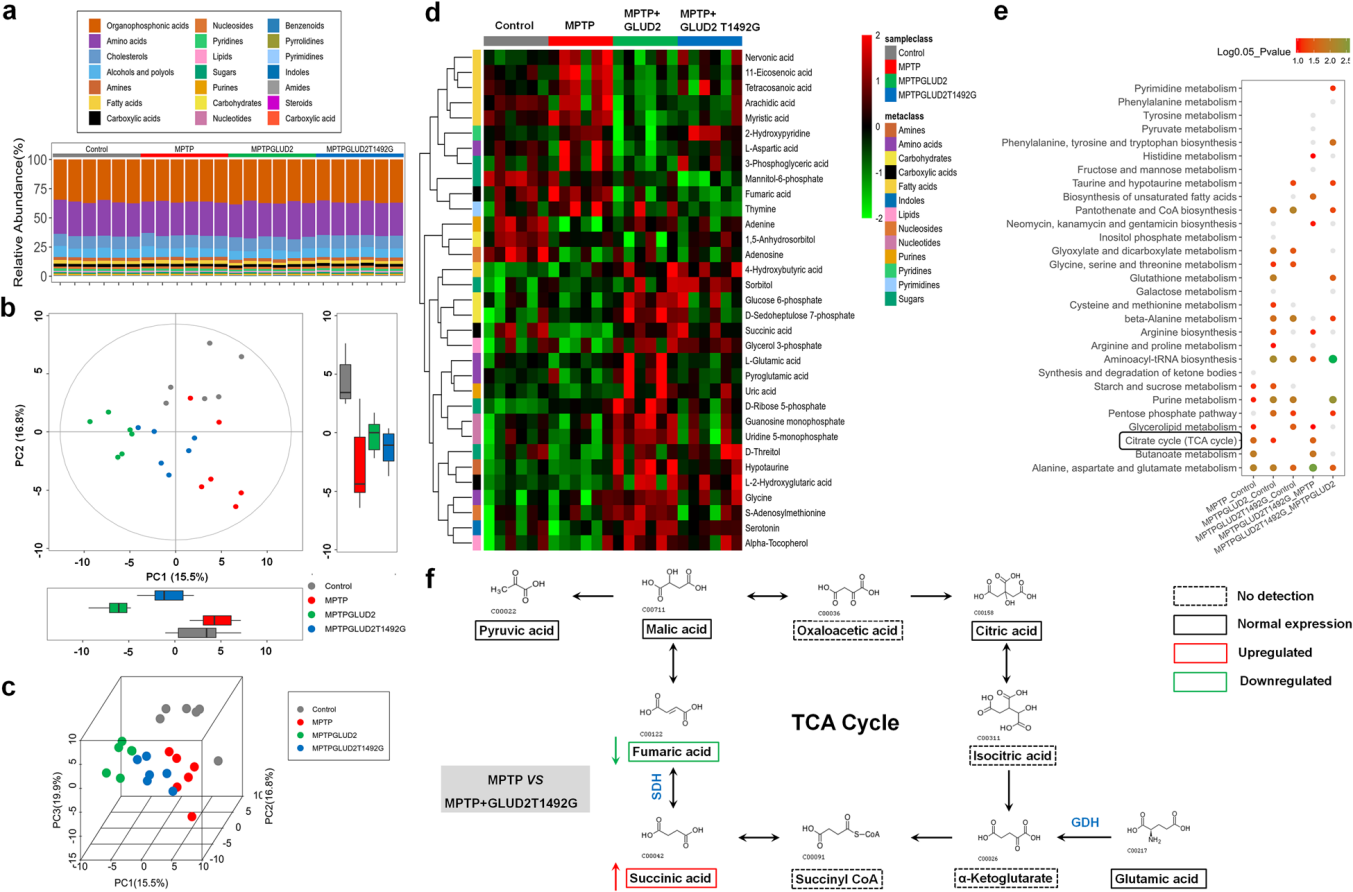
Metabolomic analysis of *GLUD2* and its mutant in the SN of MPTP-treated mice. **a** The relative abundances of representative metabolites in each sample are shown. **b** Overall metabolic profiles of samples from the AAV-GFP, MPTP + AAV-GFP, MPTP + AAV-GLUD2, and MPTP + AAV-GLUD2 T1492G groups using PCA plots. **c** Overview of metabolic profiles of all samples using 3-D PCA score plots. **d** *Z*-score plots showing the relative variations of each individual metabolite across all groups in the form of a heatmap. **e** MPEA of potential pathways involved in the differential metabolite content between the indicated groups. **f** The network of metabolites in the TCA cycle for MPTP + AAV-GFP *versus* MPTP + AAV-GLUD2 T1492G groups. Dashed boxes indicate no detection, solid boxes indicate normal expression, red boxes indicate upregulation in the MPTP + AAV-GLUD2 T1492G group compared with the MPTP + AAV-GFP group, and green boxes indicate downregulation in the MPTP + AAV-GLUD2 T1492G group compared with the MPTP + AAV-GFP group.

Given that the GDH2 protein expressed by *GLUD2* is a mitochondrial enzyme that is central to glutamate metabolism, we examined the effects of *GLUD2* mutation on nigral metabolites in MPTP-treated mice by performing GCTOF/MS (Fig. 4a and Supplementary Fig. 5a). A distinct separation of metabolites among the four groups of mice was observed in two-dimensional (2-D) boxplots and three-dimensional (3-D) principal components analysis (PCA) score plots (Fig. 4b, c), indicating significant differences in the metabolic profiles. To further evaluate these differences, we generated variable importance in projection (VIP) plots for the significant metabolites among these four groups (Supplementary Fig. 5b). Z-score plots show relative variations of each individual metabolite across all groups, which are displayed in the form of a heatmap (Fig. 4d). These results reveal metabolic differences that are specific to MPTP treatment, as well as differences that distinguish the MPTP + AAV-GLUD2 and MPTP + AAV-GLUD2 T1492G groups. To explore potential pathways involved in the differential metabolites among these four groups, we performed metabolic pathway enrichment analysis (MPEA), which revealed enrichment in metabolites of several pathways, including the citric acid cycle (TCA; also known as the CAC or Krebs cycle) (Fig. 4e).

**Fig. 5:**
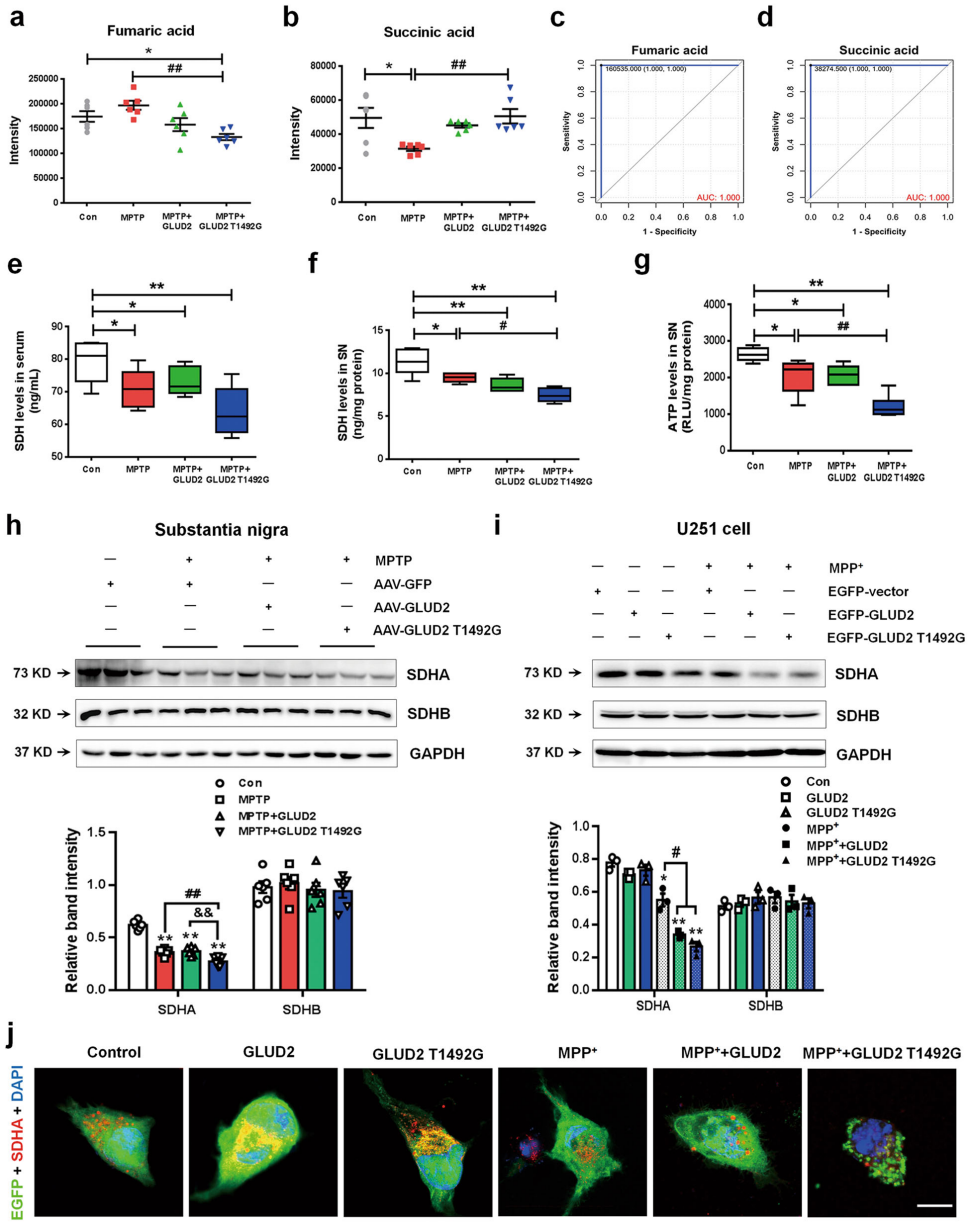
*GLUD2* mutation modulates SDH expression in MPTP-treated mice and MPP^+^-treated U251 cells. **a**, **b** Relative intensity of fumaric acid and succinic acid in the SN of AAV-GFP, MPTP + AAV-GFP, MPTP + AAV-GLUD2, and MPTP + AAV-GLUD2 T1492G groups. *n* = 6. **c**, **d** ROC analysis for fumaric acid and succinic acid. **e**, **f** SDH levels in the serum and SN of AAV-GFP, MPTP + AAV-GFP, MPTP + AAV-GLUD2, and MPTP + AAV-GLUD2 T1492G groups. *n* = 5. **g** ATP levels in the SN of AAV-GFP, MPTP + AAV-GFP, MPTP + AAV-GLUD2, and MPTP + AAV-GLUD2 T1492G groups. *n* = 6. **h**, **i** The effect of *GLUD2* or its mutant on SDHA and SDHB expression in the SN of MPTP-treated mice and MPP^+^-treated U251 cells were determined by western blotting. *n* = 6 in (**h**) and *n* = 3 in (**i**). **j** Immunofluorescent staining of EGFP and SDHA in MPP^+^-treated U251 cells expressing WT or mutant *GLUD2* (scale bars, 10 µm). Results are expressed as the mean ± SEM. ^**^p < 0.01, ^*^p < 0.05 vs. Control group. ^##^*p* < 0.01, ^#^*p* < 0.05 vs. MPTP + AAV-GFP or MPP^+^ group. ^&&^*p* < 0.01 vs. MPTP + AAV-GLUD2 group. Statistical significance was determined by one-way ANOVAs and Tukey tests for *post-hoc* comparisons.

To further evaluate the differences in TCA metabolism that are caused by MPTP treatment and *GLUD2* T1492G expression, we looked at the content of individual TCA components among the different mouse treatment groups (Fig. 4f). The results reveal that the MPTP + AAV-GLUD2 T1492G group had significantly reduced nigral fumaric acid expression and significantly increased succinic acid as compared with the levels in the AAV-GFP and MPTP + AAV-GFP groups (Fig. 5a–d). Because SDH catalyzes the oxidation of fumaric acid to succinic acid and is a key mitochondrial enzyme in mitochondrial TCA^27,28^, we also evaluated SDH levels. The results demonstrate that SDH levels in the serum and SN were significantly decreased in each of the MPTP-treated mouse groups, with the most dramatic decrease in the MPTP + AAV-GLUD2 T1492G group (Fig. 5e, f). We also examined adenosine triphosphate (ATP) levels in the SN, and the results reveal a similar trend of decrease (Fig. 5g). PD pathology is characterized by defects in mitochondrial complex I; however, low complex II/SDH activity has also been reported in PD patients^29–31^. Therefore, we further evaluated the expression of the mitochondrial complex II catalytic components SDHA and SDHB. The results demonstrate that the expression of SDHA, but not SDHB, was decreased in the SN of both MPTP-treated mice and MPP^+^-treated U251 cells, and this decrease was most significant for the GLUD2 T1492G expression group (Fig. 5h, i). Furthermore, co-staining of EGFP and SDHA in U251 cells suggests that *GLUD2* T1492G decreased SDHA expression and induced aggregate formation in the cytoplasm after MPP^+^ treatment (Fig. 5j). Collectively, these results are consistent with the possibility that *GLUD2* mutation may aggravate the effects of PD by suppressing SDHA expression, resulting in increased succinic acid levels.

### *GLUD2* mutation induces mitochondrial damage and apoptosis and represses BDNF/Nrf2 pathway expression

**Fig. 6:**
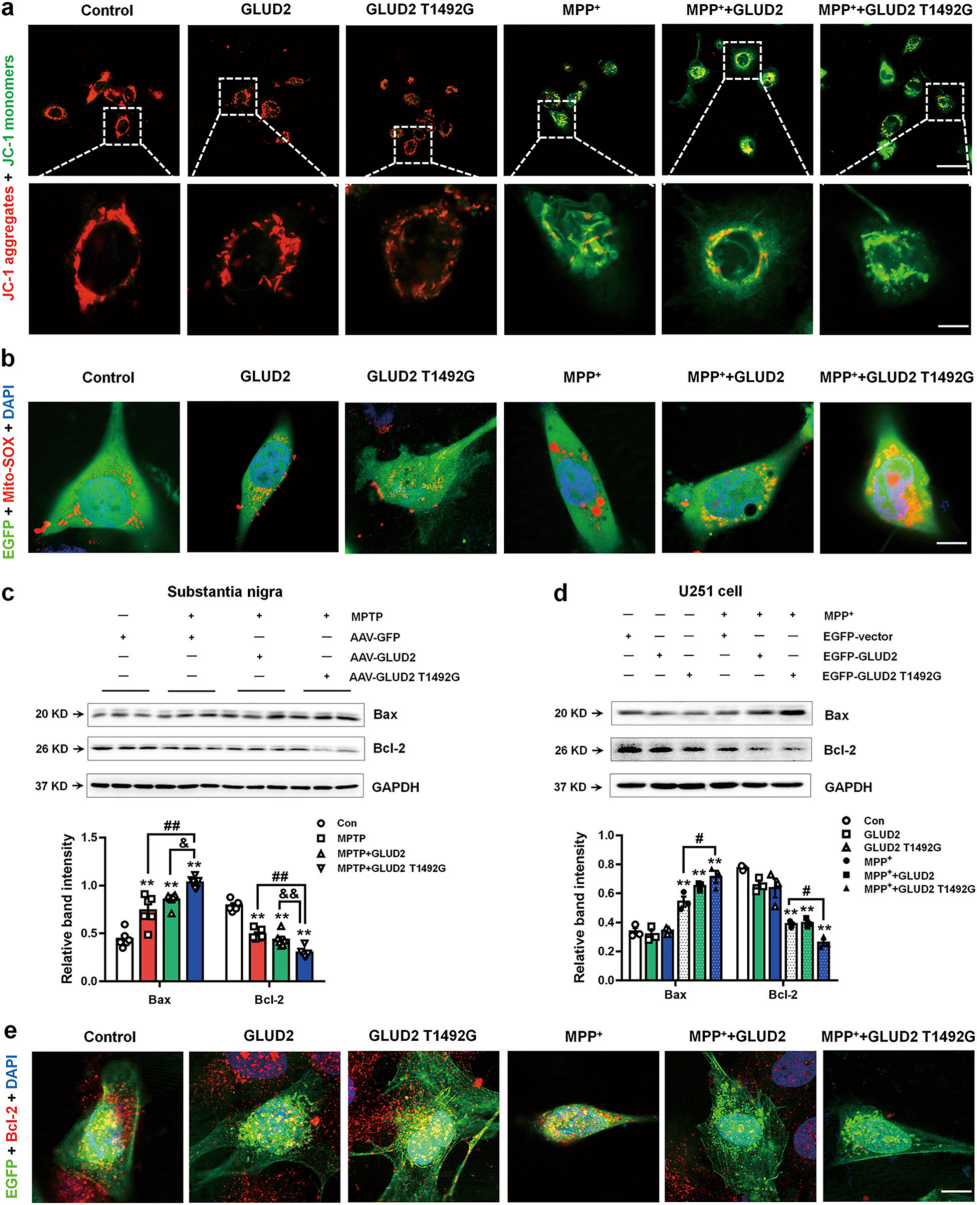
*GLUD2* mutation damages mitochondrial function in MPTP-treated mice and MPP^+^-treated U251 cells. **a** Effect of *GLUD2* or its mutant on the mitochondrial membrane potential using JC-1 fluorescent probes in control or MPP^+^-treated cells (scale bars, upper, 30 µm; lower, 5 µm). **b** Mito-SOX was used to stain live cells expressing *GLUD2* or its mutant in control or MPP^+^-treated cells (scale bars, 10 µm). **c**, **d** Effects of *GLUD2* or its mutant on Bax and Bcl-2 expression in the SN of MPTP-treated mice or in MPP^+^-treated U251 cells were determined by western blotting. *n* = 6 in (**c**) and *n* = 3 in (**d**). **e** Immunofluorescent staining of EGFP and Bcl-2 upon expression of *GLUD2* or its mutant in control or MPP^+^-treated U251 cells (scale bars, 10 µm). Results are expressed as the mean ± SEM. ^**^p < 0.01 vs. AAV-GFP group or untreated U251 cells. ^##^*p* < 0.01, ^#^*p* < 0.05 vs. MPTP + AAV^-^GFP or MPP^+^ group. ^&&^*p* < 0.01, ^&^*p* < 0.05 vs MPTP + AAV-GLUD2 group. Statistical significance was determined by one-way ANOVAs and Tukey tests for *post-hoc* comparisons.

Because our results suggest that *GLUD2* mutation may aggravate nigral DA neuron death and damage the mitochondrial TCA by reducing SDH activity, we sought to further examine its effects on mitochondrial function. Immunostaining results suggest that JC-1 aggregated within the mitochondrial matrix and formed JC-1 aggregates in the absence of MPP^+^ (indicated by red fluorescence; Fig. 6a). However, JC-1 primarily stained as monomers in MPP^+^, MPP^+^ + GLUD2 and MPP^+^ + GLUD2 T1492G groups, with almost no JC-1 aggregates remaining in the MPP^+^ + GLUD2 T1492G group (indicated by green fluorescence; Fig. 6a). These results suggest that the mitochondrial membrane potential was decreased upon MPP^+^ treatment, with further decrease in the *GLUD2* T1492G group. Next, we used Mito-SOX to label mitochondria in live cells. The Mito-SOX appeared oxidized in the MPP^+^, MPP^+^ + GLUD2 and MPP^+^ + GLUD2 T1492G groups, with the most mitochondrial damage in the MPP^+^ + GLUD2 T1492G group (Fig. 6b). As further verification, the levels of the mitochondrial-associated pro-apoptotic Bax protein were increased, while the levels of the anti-apoptotic protein Bcl-2 were decreased, both in the SN of MPTP, MPTP + AAV-GLUD2 and MPTP + AAV-GLUD2 T1492G mice (Fig. 6c), and in MPP^+^, MPP^+^ + GLUD2 and MPP^+^ + GLUD2 T1492G U251 cells (Fig. 6d). Importantly, the *GLUD2* T1492G mutant significantly increased Bax expression and decreased Bcl-2 expression in the SN of MPTP + AAV-GLUD2 T1492G *versus* MPTP + AAV-GFP mice and MPP^+^ + GLUD2 T1492G *versus* MPP^+^ U251 cells (Fig. 6c, d). The effect of *GLUD2* mutation on Bcl-2 expression was also verified by co-staining of EGFP and Bcl-2 (Fig. 6e).

**Fig. 7:**
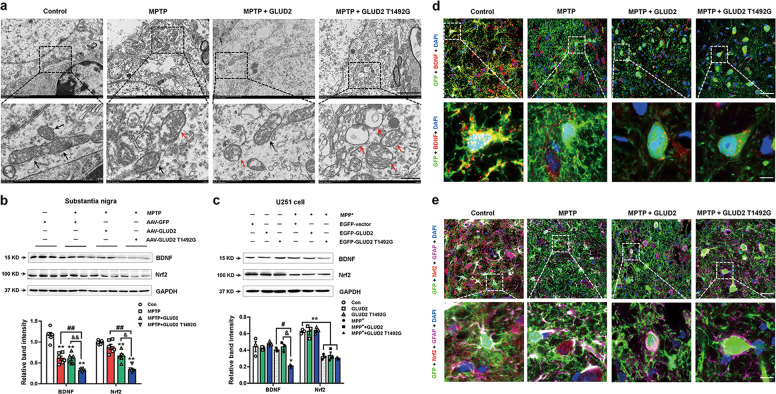
*GLUD2* mutation modulates BDNF and Nrf2 expression in MPTP-treated mice and MPP^+^-treated U251 cells. **a** Ultrastructural analysis of mitochondria in the SN of AAV-GFP, MPTP + AAV-GFP, MPTP + AAV-GLUD2, and MPTP + AAV-GLUD2 T1492G groups (scale bars, upper, 2 µm; lower, 500 nm). The black arrows indicated the normal mitochondria, and the red arrows indicated the damaged mitochondria. **b**, **c** Effect of *GLUD2* or its mutant on BDNF and Nrf2 expression in the SN of MPTP-treated mice or in MPP^+^-treated U251 cells was determined by western blotting. *n* = 6 in (**b**) and *n* = 3 in (**c**). **d** Immunofluorescent staining of GFP and BDNF in the SNpc of AAV-GFP, MPTP + AAV-GFP, MPTP + AAV-GLUD2, and MPTP + AAV-GLUD2 T1492G groups (scale bars: upper, 40 µm; lower, 8 µm). **e** Immunofluorescent staining of GFP, GFAP, and Nrf2 in the SNpc of AAV-GFP, MPTP + AAV-GFP, MPTP + AAV-GLUD2, and MPTP + AAV-GLUD2 T1492G groups (scale bars, upper, 40 µm; lower, 8 µm). Results are expressed as the mean ± SEM. ^**^p < 0.01 vs. AAV-GFP or untreated U251 cell group. ^##^*p* < 0.01, ^#^*p* < 0.05 vs. MPTP + AAV^-^GFP or MPP^+^ group. ^&&^*p* < 0.01, ^&^*p* < 0.05 vs. MPTP + AAV-GLUD2 group. Statistical significance was determined by one-way ANOVAs and Tukey tests for *post-hoc* comparisons.

To further verify the effect of *GLUD2* mutation in inducing mitochondrial damage, we observed the mitochondria at the ultrastructural level by transmission electron microscopy (TEM). There were significantly fewer intact mitochondria in the SN of MPTP-treated mice, as compared with AAV-GFP group (Fig. 7a and Supplementary Fig. 6a). The numbers of mitochondria showed no obvious differences between MPTP + AAV-GFP and MPTP + AAV-GLUD2 T1492G groups (Supplementary Fig. 6a); however, *GLUD2* T1492G-induced distinct mitochondrial morphological changes in the SN of MPTP-treated mice, such as mitochondrial swelling, mitochondrial cristae disappearance, and mitochondrial fragmentation (indicated by the red arrows in Fig. 7a). We also determined that the expression of the neurotrophin family growth factor BDNF, and the signaling mediator Nrf2 was significantly decreased in the SN of MPTP + AAV-GFP, MPTP + AAV-GLUD2 and MPTP + AAV-GLUD2 T1492G mice; the reduction was most obvious for the MPTP + AAV-GLUD2 T1492G group (Fig. 7b). Consistently, in U251 cells, BDNF expression was decreased in the MPP^+^ + GLUD2 T1492G group as compared with the MPP^+^ group, and Nrf2 expression was decreased in the MPP^+^, MPP^+^ + GLUD2 and MPP^+^ + GLUD2 T1492G groups (Fig. 7c). Co-staining of GFP, GFAP, and BDNF/Nrf2 in the SNpc supports our findings (Fig. 7d, e). These effects appear to be specific to MPTP induction, as *GLUD2* and its mutant showed no obvious effects on Bax, Bcl-2 or BDNF expression in the SN of untreated mice (Supplementary Fig. 6b). Therefore, our results support the role of *GLUD2* mutation in exacerbating reduced BDNF/Nrf2 signaling, mitochondrial damage, and cell death in MPTP-treated mice.

## Discussion

**Fig. 8:**
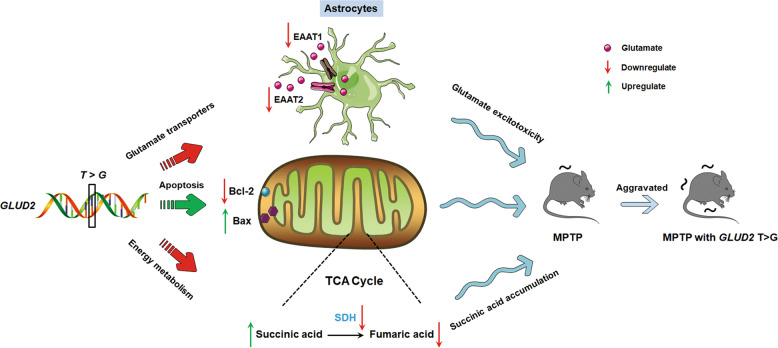
Schematic models showing the underlying mechanism by which *GLUD2* T1492G variant modifies disease onset in the MPTP-induced PD animal model. In this study, the *T1492G* variant of *GLUD2* decreases glutamate transporters expression and function, induces apoptosis by decreasing Bcl-2 and increasing Bax expression, and reduces energy metabolism by decreasing succinate dehydrogenase activity. These effects cause glutamate excitotoxicity and succinic acid accumulation. Consequently, *GLUD2* variant aggravates nigral dopaminergic neuron death and exacerbates movement deficits in MPTP-induced PD mice. In the schematic model, red arrows mean downregulate and green arrows mean upregulate.

In the present study, for the first time, we determined that a rare *GLUD2* mutation may aggravate motor function and nigral DA neuron death in MPTP-treated mice. We also revealed a possible mechanism that may involve *GLUD2* mutant-associated damage of mitochondrial function via targeting of SDH, reduced glutamate transporters expression and function, and increased apoptosis, potentially caused by a decrease in BDNF/Nrf2 signaling (Fig. 8).

In this study, we sought to explore the effects and mechanisms of *GLUD2* mutation in astrocytes in MPTP-treated mice. Our results are consistent with the possibility that *GLUD2* mutation induces glutamate excitotoxicity by reducing the expression and function of astroglial glutamate transporters (EAAT1 and EAAT2) in MPTP-treated mice. Previously, we and other groups have reported that EAAT1 and EAAT2 are decreased in PD animal models and PD patients, and glutamate excitotoxicity mediated by dysfunctional glutamate transporters in astrocytes contributes to the pathogenesis of PD^25,32–34^. Notably, deletion of astroglial EAAT2/GLT-1 in the SNpc caused DA neuron death and behavioral impairment in rodents^32,34^. Here, we found that mutated *GLUD2* decreased EAAT1 and EAAT2 expression and reduced glutamate uptake in the SN of MPTP-treated mice and the MPP^+^-treated glioma cell line U251. We conclude that several factors may contribute to the decreased expression and function of these transporters. First, mitochondrial oxidative phosphorylation is required to maintain the functional Na^+^ and K^+^ gradient, which is utilized by glutamate transporters to take up glutamate and generate energy^35^. EAAT2 has been reported to be co-compartmentalized with glycolytic enzymes and mitochondria^36^. In this study, we demonstrated that the *GLUD2* T1492G-induced mitochondrial energy defect also affects glutamate transporter function and increases Bax and decreases Bcl-2 expression, suggesting that *GLUD2* T1492G may induce astroglial apoptosis, which may further decrease glutamate transporter expression. Interestingly, MPEA results revealed that the differential metabolites were enriched in the glutamate metabolism signaling pathway (especially between the MPTP + AAV-GFP and MPTP + AAV-GLUD2 T1492G groups), which is consistent with our results suggesting that *GLUD2* mutation may decrease glutamate transporters and induce excitotoxicity. Therefore, glutamate excitotoxicity mediated by reduced glutamate transporter expression and function may, in turn, contribute to astroglial *GLUD2* mutant-aggravated nigral cell death in MPTP-treated mice.

The protein encoded by *GLUD2* catalyzes the reversible inter-conversion of glutamate to α-ketoglutaric acid and ammonia, and *GLUD2*-mediated glutamate oxidation is associated with the Krebs cycle and energy metabolism^37,38^. Here, we identified a new role of *GLUD2* in mitochondrial energy metabolism by targeting SDH, an enzyme of the mitochondrial complex II. The electron transport chain (ETC) consists of four enzymes—mitochondrial complex I–IV—and generally complex I is a major entry point of the ETC. Deficiencies in complex I have been reported in the SNpc of PD patients’ brain samples^29^. In addition, neurotoxins, including MPTP and its active metabolite MPP^+^, can induce PD models by inhibiting complex I, which mimics main pathological features of the disease^39,40^. However, complex II/SDH is another entry point of the ETC for reducing equivalents, and several studies have also reported mitochondrial complex II defects and lower activity in PD patients^30,31^. SDH oxidizes fumaric acid to succinic acid and is a key enzyme in the mitochondrial TCA^28^. SDHA and SDHB are the major catalytic subunits of mitochondrial complex II, with the SDHA subunit catalyzing the reaction by transferring electrons to SDHB via the electron transport coenzyme, flavin adenine dinucleotide^41^. In addition, SDH can provide electrons to complex I via reverse transport. Because SDH links the TCA cycle and the respiratory chain, it is also responsible for ATP production, though much less than complex I^42^, and increasing evidence suggests that complex II is a key modulator in neuroprotection, possibly attributed to its close correlation with rates of NADH-driven electron flow^43,44^. Moreover, inhibition of SDH leads to succinic acid accumulation in the cell, which may also affect ATP production under certain conditions^45^. Enhancing complex II activity has been revealed to selectively rescue complex I inhibition-mediated mitochondrial respiratory impairment^46^. Thus, in this study, though MPTP is mainly responsible for the ATP reduction and behavioral disorder in mice, decrease of complex II/SDH activity by *GLUD2* mutation may further impede energy metabolism and ATP production in MPTP mice, and this may aggravate the movement disorder in the MPTP mouse model of PD.

In this study, we also found that *GLUD2* mutation affects the anti/pro-apoptotic protein ratio in PD mice and a cellular model. The pro-apoptotic Bax and anti-apoptotic Bcl-2 proteins reside at the mitochondria and are reported to mediate the intrinsic apoptosis pathway by controlling mitochondrial outer membrane integrity^47^. Moreover, these proteins are proposed to control the opening of the mitochondrial permeability transition pore, which is located between the outer and inner mitochondrial membranes^48^. Thus, we conclude that *GLUD2* T1492G induction of the dysfunctional mitochondrial function may involve alteration of Bax and Bcl-2 expression, which would further damage astrocytes. We also demonstrated that the BDNF/Nrf2-signaling pathway may be responsible for the induction of nigral cell death by *GLUD2* T1492G. BDNF is an important metabolic linker between astrocytes and neurons that regulates formation of neuronal synapses, glucose and lipid metabolism in astrocytes^49,50^. Nrf2 is a master regulator for the cellular defense against oxidative stress, and it can be activated by BDNF^51^. BDNF-mediated Nrf2 activation in astrocytes has been demonstrated to protect DA neurons from death in PD^52^. Furthermore, SDHA engages in inflammatory mitochondrial retrograde signaling via activation of Nrf2 in immune cells^53^. BDNF also has been reported to protect neurons against apoptotic cell death caused by inhibition of mitochondrial complex II^54^. Therefore, BDNF/Nrf2 signaling is likely to be important in maintaining the function of mitochondrial complex II formed by SDH. In this study, we demonstrated that *GLUD2* T1492G decreases both BDNF and Nrf2 expression in MPTP-treated mice, suggesting that this pathway may also be responsible for nigral cell death. Thus, the Bcl-2/Bax and BDNF/Nrf2 pathways may both be closely associated with *GLUD2* mutant-induced mitochondrial damage.

As mentioned above, rodents and other mammals express only one GDH isoform, GDH1; however, human and apes also possess another isoform, GDH2^15^. Previously, to examine the physiological effects of hGDH2 in rodent brains, several groups have generated hGDH2-expressing transgenic mice by inserting a bacterial artificial chromosome containing the human *GLUD2* gene into mice^20,55^. They found that hGDH2 did not affect glutamate levels in mice, but it affected carbon flux during early brain development^55^. Moreover, hGDH2 promotes TCA cycle capacity and oxidative metabolism of glutamate during glucose deprivation in astrocytes from hGDH2-expressing transgenic mice^20^. To investigate the effects of T1492G variant in *GLUD2* in PD mice, we generated an AAV-virus carrying WT and mutated *GLUD2* with astrocytes marker. Therefore, we believe that it is scientifically sound to study the glutamate-associated function of this gene in the PD mouse model.

Although the T1492G variant in *GLUD2* has never been reported in PD patients since 2010, this rare variant is of interest for several reasons. First, glutamate excitotoxicity is a predominant factor in PD pathogenesis; previous studies have mainly focused on the astroglial glutamate transporters and have verified the important role of glutamate transporters in PD^25,33,34^. However, glutamate transporters are mainly involved in glutamate uptake from the synaptic cleft, rather than the glutamate metabolism. GDH2 is vital not only for catabolizing glutamate to reduce excitotoxicity, but also for converting it into α-ketoglutarate to boost ATP generation in mitochondria. Thus, this gene may play an important role in glutamate excitotoxicity in PD. Second, GDH2 expression is mainly in the brain and its gene is located on the X chromosome; therefore, this could explain why males are more vulnerable than females to PD, with less concentration of glutamate and more ATP production. Thus, GDH2 could be critical in preventing excitotoxicity-related neurological diseases, such as PD. Lastly, because *GLUD2* only exists in human and apes, rather than rodents, we employed an approach (AAV-virus or transgenic mice) to explore how this mutated gene works in a rodent model. In the present study, we found mutated *GLUD2* decreased astroglial glutamate transporter expression and function, and we also revealed a possible mechanism that may be involved in astroglial mitochondrial metabolism. Nevertheless, evaluation of the genetic variant in the *GLUD2* region in larger cohorts of PD patients is necessary to confirm its function in the future.

Recently, succinic acid accumulation via SDH and elevation of mitochondrial membrane potential have been demonstrated to drive mitochondrial reactive oxygen species (ROS) production^56,57^. Furthermore, accumulation of succinic acid in the cell has been demonstrated to be the main instigator of ROS generation^58^. Generally, superoxide production is associated with high membrane potential and typically, reduction in the ubiquinone pool^59^. However, under certain pathological conditions, such as mitochondrial disorders associated with dysfunctional respiratory chain components, lower membrane potential and decreased activity of the respiratory chain are observed with a simultaneous increase in ROS production^60,61^. In addition, MPTP or MPP^+^ is thought to reduce mitochondrial membrane potential and increase ROS production^62–65^. Thus, the inverse correlation between membrane potential and ROS production observed in this study may be due to a combination of succinic acid accumulation via a *GLUD2* mutant-associated decrease in SDH activity and the MPTP mouse or MPP^+^ cellular model that we used.

In addition to the effect of *GLUD2* expression in inducing multiple changes in the SN related to mitochondrial damage and glutamate metabolism, we demonstrated that *GLUD2* causes decreased TH expression in MPTP-treated mice. However, the motor deficits were more obvious for the *GLUD2* mutant and were not observed in the absence of MPTP treatment. This may explain why *GLUD2* T1492G contributes to, rather than induces, the disease phenotype of PD patients^24^. In addition, the lack of effect of the T1492G variant in *GLUD2* for female PD patients may be explained by cross regulation by estrogens^24^. Our attempts at evaluating the effects of this variant in female PD animals proved difficult because female C57/BL mice are highly sensitive to MPTP, with nearly all mice dying after MPTP administration, which is consistent with a previous report^66^. Thus, another PD animal model may verify the potential relationship between estrogen and the *GLUD2* T1492G mutant in future studies.

In summary, we generated and injected virus expressing *GLUD2* and its mutant in the SNpc of MPTP-treated mice. We found that the *GLUD2* T1492G mutant exacerbates the movement deficiency and nigral DA neuron death in MPTP-treated mice. The underlying mechanism may involve *GLUD2* mutant-reduced glutamate transporter expression and function, damaged mitochondrial function via decreasing SDH activity, and induced apoptosis via downregulated BDNF/Nrf2 signaling in MPTP-treated mice and MPP^+^-treated U251 cells. Collectively, our findings support the possibility that the T1492G variant of *GLUD2* may modify disease onset in male PD patients.

## Materials and methods

### Reagents

MPP^+^ and MPTP were purchased from Sigma-Aldrich (St. Louis, MO, USA). Anti-TH (F-11, sc-25269), dopamine transporter (DAT, sc-32258), GDH2 (sc-293459), GDH1 (sc-515542), and pyruvate dehydrogenase E1α (PDH-E1α) (sc-377092) antibodies were purchased from Santa Cruz Biotechnology (Dallas, TX, USA). Anti-BDNF (ab108319), SHDA (ab137040), and SDHB (ab178423) antibodies were purchased from Abcam (Cambridge, MA, USA). Anti-GFAP (#80788), NR2A (#4205), GluA1 (#13185), Bax (#14796), and GluA2 (#13607) antibodies were purchased from Cell Signaling Technology (Danvers, MA, USA). EAAT1 (20785-1-AP), EAAT2 (22515-1-AP), Nrf2 (16396-1-AP), Bcl-2 (16396-1-AP), and GAPDH (60004-1) antibodies were purchased from Proteintech Group (Rosemont, IL, USA). DyLight 488 goat anti-mouse IgG (H + L) (70-GAM4882) and DyLight 594 goat anti-rabbit IgG (H + L) (70-GAR5942) were purchased from Multi Sciences (Hangzhou, China). Horseradish peroxidase (HRP)-labeled goat anti-rabbit IgG and HRP-labeled goat anti-mouse IgG were purchased from Beyotime Biotechnology (Shanghai, China). Mito-SOX™ Red Mitochondrial Superoxide Indicator was purchased from Thermo Fisher Scientific (Waltham, MA, USA).

### Animals

All animal experimental procedures were performed according to the National Institute of Health guidelines on the care and use of animals (NIH Publications No. 8023, revised 1978) and were approved by Guangzhou Medical University Animal Care and Use Committee. Eight-week-old healthy male C57BL/6 mice, body mass (24 ± 2) g, were purchased from SPF Biotechnology Co., Ltd (Beijing, China) and housed in the animal laboratory of Guangzhou Medical University with a 12 h dark/light cycle at ambient temperature (22 ± 1) °C and relative humidity (60 ± 5)%. Mice were allocated randomly to experimental groups and those with movement deficiency were excluded from the study. We calculate the samples size based on experience with the respective tests, variability of the assays and inter-individual differences within groups.

### Glioma U251 cell culture

U251 cells were purchased from American Type Culture Collection (Manassas, VA, ATCC) and were cultured in basic Dulbecco’s Modified Eagle Medium (GIBCO, Carlsbad, CA, USA) containing 8% fetal bovine serum (GIBCO, Carlsbad, CA, USA), 2 U/mL penicillin (Beyotime Biotechnology), and 2 mg/mL streptomycin (Beyotime Biotechnology) at 37 °C and 5% CO_2_ in an incubator.

### Overexpression of *GLUD2* and its mutant in U251 cells

Enhanced green fluorescent protein (EGFP)-vector, EGFP-GLUD2, and EGFP-GLUD2 T1492G plasmids were provided by Dongze Biotechnology Co., Ltd (Guangzhou, China). U251 cells were seeded at a density of 1.0 × 10^6^ cells/well at 50% confluency. Lipofectamine™ 3000 reagent (Invitrogen) or DNA was diluted in Opti-MEM™ medium and was then combined with the plasmids. The mixture was incubated for 15 min at RT and was then added to the cells. Two days later, the cells were treated with 500 µM MPP^+^ for 24 h, and then western blotting and immunofluorescence assays were performed.

### Immunocytochemical staining

Immunocytochemical staining was performed according to our previous work^34^. Briefly, cells were incubated with primary antibodies overnight at 4 °C. The next day, they were washed in PBS and incubated with fluorescent-labeled secondary antibody for 1 h at 37 °C. Then, 4′,6-diamidino-2-phenylindole (DAPI) was used to stain cellular nuclei, and images were scanned under a confocal laser-scanning microscope (SP8; Leica, Hamburg, Germany).

### Measurement of the mitochondrial membrane potential

The mitochondrial membrane potential was analyzed according to the manufacturer’s instructions (Beyotime Biotechnology). Briefly, after *GLUD2* WT or mutant overexpression and MPP^+^ treatment for 72 h, U251 cells were washed once in PBS and incubated with JC-1 staining solution at 37 °C for 20 min. After two washes with JC-1 staining buffer, cells were added to the culture medium, and images were scanned under a confocal laser-scanning microscope (SP8; Leica).

### Mitochondrial superoxide imaging of live cells

Mito-SOX™ Red Mitochondrial Superoxide Indicator was used to stain live cells according to the manufacturer’s instructions (Thermo Fisher Scientific). Briefly, after *GLUD2* WT or mutant overexpression and MPP^+^ treatment for 72 h, U251 cells were incubated with 5 µM red fluorescent dye-based Mito-SOX™ at 37 °C for 10 min, and images were scanned under a confocal laser-scanning microscope (SP8; Leica).

### Stereotaxic injection of AAV-GLUD2 WT or mutant virus into the SNpc

The AAV-GLUD2 and AAV-GLUD2 T1492G viruses were generated by ligating annealed oligonucleotides encoding *GLUD2* and *GLUD2* T>G into the *Xba* I/*Eco* RI site of the AAV2/9-GFAP-3Flag-GFP-polyA (PSE) overexpression vector, which replaces the GFP-encoding sequence but maintains the Flag tag. The viruses were constructed to express *GLUD2* and *GLUD2* T>G via the GFAP promoter, and they were packaged by Sunbio Medical Biotechnology (Shanghai, China). Control virus AAV-GFP with the GFAP promoter was also provided by Sunbio Medical Biotechnology (Shanghai, China). AAV-GFP (viral: 5.94 × 10^12^ particles mL^–1^), AAV-GLUD2 (viral: 9.20 × 10^12^ particles mL^–1^), or AAV-GLUD2 T1492G (viral: 8.23 × 10^12^ particles mL^–1^) were stereotaxically injected into the SNpc as previously described^34^. Mice were anesthetized and placed in a stereotaxic frame. The viruses in 0.5 µL vol were delivered into the bilateral SNpc at the target site, as reported previously (Bregma AP, −3.0 mm, ML, ±1.3 mm, DV, −4.7 mm)^33^. The syringes were left in place for 5 min before being slowly withdrawn from the brain. To determine the effects of *GLUD2* or its mutant in untreated mice and MPTP-induced mice, we divided the mice into AAV-GFP, MPTP + AAV-GFP, MPTP + AAV-GLUD2, and MPTP + AAV-GLUD2 T1492G groups. Three weeks after stereotaxic injection of the AAVs, mice in the MPTP + AAV-GFP, MPTP + AAV-GLUD2, and MPTP + AAV-GLUD2 T1492G groups were intraperitoneally injected with MPTP (25 mg/kg), twice a week, for another 5 weeks, while mice in the AAV-GFP group were injected with saline. After completion of behavioral tests, mice in each group were sacrificed for sample collection.

### Behavioral tests

#### Open-field test

Open-field testing was performed as previously described^67^. Mice were placed in the middle of the central area and observed in dim light for 15 min. A video tracking system, EthoVisione XT software (Beijing, China), was used to record the distance and time of movement in the open field. The test apparatus was thoroughly cleaned between tests with different animals.

#### Grip strength test

A grip strength tester (Ugo Basile SRL, Gemonio, VA, Italy) was used to measure neuromuscular strength as previously described^68^. Grip strength was digitally recorded and measured in grams (g).

#### Rotarod test

Mice were trained 3 days at a speed of 10 rpm/s for 5 min prior to testing as previously described^68^. On the test day, the mice were placed on an accelerating rotating cylinder (from 4 to 40 rpm/s) and their time to falling was recorded.

#### Pole-climbing test

Before the test, mice were conditioned in the behavior room for 30 min. The pole consisted of a 75-cm metal rod with a diameter of 9 mm that was wrapped in bandage gauze^69^. Mice were placed on top of the pole head upward, and they were trained for 2 days before testing. The maximum cut-off time to stop testing and recording was 60 s. The total time (in sec) that it took the mice to descend to the bottom was recorded.

#### Grasping test

The mice were suspended by the forelimbs on a horizontal wire with a diameter of 1 mm for 10 s, and their abilities to grasp a wire with their hind legs was measured. The scoring was as follows: The mouse grasped the wire with both hind legs, 3; the mouse grasped the wire with only one hind limb, 2; the mouse could not grasp the wires with either hind limb, 1; the mouse dropped, 0. Results were from three average measurements.

### Metabolomics analysis of SN samples

#### Metabolite extraction

Tissues (100 mg) were ground in liquid nitrogen, and homogenates were resuspended in prechilled 80% methanol and 0.1% formic acid with thorough vortexing. The samples were incubated on ice for 5 min and then were centrifuged at 15,000 rpm and 4 °C for 5 min. The supernatant was diluted to a final concentration of 53% methanol in liquid chromatography-mass spectrometry (LC–MS) grade water. Samples were subsequently transferred to fresh Eppendorf tubes and then were centrifuged at 15,000 × *g* at 4 °C for 10 min. Finally, the supernatants were analyzed by the LC–MS/MS system.

#### UHPLC–MS/MS analysis

LC–MS/MS analysis was performed using a Vanquish UHPLC system (Thermo Fisher) coupled with an Orbitrap Q Exactive series mass spectrometer (Thermo Fisher). Samples were injected into a Hyperil Gold column (100 × 2.1 mm, 1.9 μm) using a 16 min linear gradient at a flow rate of 0.2 mL/min. The eluents for the positive polarity mode were A (0.1% formic acid in water) and B (methanol). The eluents for the negative polarity mode were A (5 mM ammonium acetate, pH 9.0) and B (methanol). The solvent gradient was set as follows: 2% B, 1.5 min; 2–100% B, 12.0 min; 100% B, 14.0 min; 100–2% B, 14.1 min; 2% B, 17 min. The Q Exactive series mass spectrometer was operated in positive/negative polarity mode with a spray voltage of 3.2 KV, capillary temperature of 320 °C, sheath gas flow rate of 35 arb, and aux gas flow rate of 10 arb.

#### MS data analysis

Raw data files generated by UHPLC–MS/MS were processed using the Compound Discoverer 3.1 (CD3.1, Thermo Fisher) to perform peak alignment, peak selection, and quantitation for each metabolite. The main parameters were set as previously described^67^. The metabolites were annotated using the KEGG database (http://www.genome.jp/kegg/), HMDB database (http://www.hmdb.ca/), and Lipidmaps database (http://www.lipidmaps.org/). PCA and Partial least squares discriminant analysis were performed on the metaX platform. Univariate analysis (T-testing) was used to calculate the statistical significance (*P* value). Metabolites with VIP > 1, *P* value < 0.05, and fold change ≥ 2 or FC ≤ 0.5 were considered differential metabolites. Volcano plots were used to filter metabolites of interest based on the Log_2_ (fold change) and -Log_10_ (*P* value).

### Western blotting

Western blotting was performed as described in our previous study^70^. Samples of SN and striatum were subjected to electrophoresis by SDS-PAGE and then transferred to polyvinylidene fluoride (PVDF) membranes. The PVDF membranes were incubated with primary antibody overnight at 4 °C, and then with HRP-labeled secondary antibodies at room temperature (RT) for 1 h. Chemiluminescence was visualized on the GeneGnome XRQ Chemiluminescence imaging system (Gene Company, Hong Kong, China). Image J software was used to analyze the optical density of bands.

### Immunohistochemistry and immunofluorescence assays

Immunohistochemistry and immunofluorescence assays were performed as described previously^68^. Briefly, embedded mouse brains were cut into 15-μm sections with a freezing microtome (Leica), and the slices were incubated with corresponding primary antibodies overnight at 4 °C. For the immunohistochemistry assay, brain slices were incubated with a secondary antibody labeled with biotin and stained with diaminobenzidine. Images were scanned under a light microscope (Leica). For the immunofluorescent assay, brain slices were incubated with fluorescent-labeled secondary antibodies and DAPI to stain nuclei. Images were scanned under a confocal laser-scanning microscope (SP8; Leica). Quantitative analysis was performed using the Image-Pro Plus 6.0 photogram analysis system (IPP 6.0, Media Cybernetics, Bethesda, MD, USA).

### Transmission electron microscopy (TEM)

The ultrastructural morphology of mitochondria was analyzed by TEM as described previously^68^. Briefly, tissues were fixed in electron microscope fixation solution at 4 °C for 2–4 h and further fixed with 1% osmium in 0.1 M phosphate buffer PB (pH 7.4) at RT for 2 h. The tissues were dehydrated with an increasing gradient of alcohol followed by incubation in 100% acetone for 15 min. Then the tissues were embedded with 812 embedding agent (SPI-Pon 812 Epoxy Resin Monomer; SPI, Shanxi, China). The samples were sliced into ultra-thin sections of 70 nm with an ultra-thin slicer (Leica), and then the sections were double-stained with uranium lead and dried overnight at RT. The photographs were imaged and analyzed by TEM (HT7700; Hitachi, Tokyo, Japan).

### ATP detection assay

ATP detection assays were performed using ATP assay kits (Beyotime Biotechnology, Shanghai, China) according to the manufacturer’s instructions. In brief, SN samples were lysed with 200 µL lysis buffer via ultrasound and centrifuged at 12,000 rpm for 5 min to obtain supernatant. Samples were mixed with 100 µL ATP detection working buffer and measured on a plate-reading luminometer. The concentration of ATP is expressed as relative fluorescence units/mg of protein.

### Enzyme-linked immunosorbent assay (ELISA)

Mouse serum and nigral SDH concentrations were measured with ELISA kits (AndyGene Biotechnology Co., Ltd, Beijing, China) according to the manufacturer’s instructions. Nigral samples were homogenized in ice-cold PBS containing protease inhibitor by sonication with an ultrasonic cell disrupter. The homogenates were then centrifuged for 5 min at 5000 × *g* to retrieve the supernatants. Serum and nigral samples were added to SDH antibody-coated plates, and the plates were incubated at 37 °C for 30 min. Afterward, horseradish peroxidase (HRP)-labeled secondary antibody was added to each well, and the plates were incubated at 37 °C for an additional 30 min. Then, chromogenic agents A and B were added to each well and the plates were incubated at 37 °C for 15 min. Stop solution was added, and the OD values were obtained by Multiscan Spectrum (PerkinElmer) at 450 nm.

### Statistical analysis

Statistical tests were performed using GraphPad Prism 8.0 (GraphPad Software, La Jolla, CA) via one-way analysis of variance (ANOVA) followed by the Tukey’s *post-hoc* test for multiple comparisons. All data are expressed as the mean ± standard error of the mean (SEM), with the statistical significance level set at *p* < 0.05.

## Supplementary information

Supplemental Methods

Supplementary Figure Legends

Supplementary Figure 1

Supplementary Figure 2

Supplementary Figure 3

Supplementary Figure 4

Supplementary Figure 5

Supplementary Figure 6
