# Supplementary material for: Functional validation of a human GLUD2 variant in a murine model of Parkinson’s disease
Source: Cell Death Dis. 2020 Oct 22;11(10):897. doi: 10.1038/s41419-020-03043-2 (PMC7582183; doi:10.1038/s41419-020-03043-2)
Supplement: Supplementary file 1 — Supplemental Methods [file 41419_2020_3043_MOESM1_ESM.docx]

**Supplementary Methods**

**Golgi staining**

Golgi staining was performed using the FD Rapid Golgi Stain™ Kit (FD Neuro Technologies, Inc., MD, USA) as previously described[^1^](#_ENREF_1)^,^[^2^](#_ENREF_2). In brief, brains were removed, immersed in impregnation solution, and mixed with equal volumes of Solutions A and B at RT for 2 weeks in the dark. Then the brains were transferred into Solution C and stored at RT in the dark for 72 h. Brain sections were cut at 150 μm on a cryostat at -20°C, and rinsed twice in double distilled water. Then the sections were placed in a mixture consisting of one part of Solution D, one part of Solution E, and two parts of double distilled water for 10 min. After rinsing twice, the sections were dehydrated in 50%, 75% and 95% ethanol. The Golgi-stained neurons and dendritic segments in striatum were imaged with the Axio Imager microscope (Carl Zeiss), and the numbers of spines per 10 µm were counted and quantified with blinding.

**L-[^3^H]-glutamic acid uptake assay**

L-[^3^H]-Glutamic acid uptake assays were performed as we stated previously[^3^](#_ENREF_3). U251 cells were incubated with 0.4 mCi L-[^3^H]-Glutamic acid (specific activity 12.9 Ci/mmol) in choline solution (150 mM choline chloride, 5 mM KP_i_, pH 7.4, 0.5 mM MgSO_4_ and 0.3 mM CaCl_2_) at room temperature for 10 min. Then ice-cold NaCl solution (150 mM NaCl, 5 mM KP_i_, pH 7.4, 0.5 mM MgSO_4_ and 0.3 mM CaCl_2_) was used to terminate the reaction. Liquid scintillation counting was used to measure the radioactivity.

**Determination of glutamate uptake in the synaptosomes**

Glutamate uptake in the synaptosomes of the substantia nigra (SN) was performed as we stated previously[^4^](#_ENREF_4). The crude synaptosomes of substantia nigra were obtained by homogenizing in 0.32 M sucrose solution (0.32 M sucrose, 5 mM Hepes, pH 7.4). Then it was incubated with 1 mCi L-[^3^H]-glutamic acid and following resuspension in Kreb's buffer (127 mM NaCl, 3.73 mM KCl, 1.8 mM CaCl_2_, 1.18 mM KH_2_PO_4_, 20 mM NaHCO_3_, 2 mM ATP, 2 g/l D-glucose, pH 7.4). Ice-cold Kreb's buffer was used to terminate the reaction. Liquid scintillation counting was used to measure the radioactivity. Data are from three separate experiments performed in triplicate and are expressed as cpm/mg protein/min.

**Statistical Analysis**

Statistical tests were performed using GraphPad Prism 8.0 (GraphPad Software, La Jolla, CA) via one-way analysis of variance (ANOVA) followed by the Tukey’s *post-hoc* test for multiple comparisons. All data are expressed as the mean ± standard error of the mean (SEM), with the statistical significance level set at *p* < 0.05.

**References**

1. Toy, W. A. *et al.* Treadmill exercise reverses dendritic spine loss in direct and indirect striatal medium spiny neurons in the 1-methyl-4-phenyl-1,2,3,6-tetrahydropyridine (MPTP) mouse model of Parkinson's disease. *Neurobiol Dis* **63**, 201-209 (2014).

2. Wang, H., Lewsadder, M., Dorn, E., Xu, S. & Lakshmana, M. K. RanBP9 overexpression reduces dendritic arbor and spine density. *Neuroscience* **265**, 253-262 (2014).

3. Zhang, Y. *et al.* Regulation of glutamate transporter trafficking by Nedd4-2 in a Parkinson's disease model. *Cell death & disease* **8**, e2574 (2017).

4. Zhang, Y. L. *et al.* Ginsenoside Rb1 confers neuroprotection via promotion of glutamate transporters in a mouse model of Parkinson's disease. *Neuropharmacology* **131**, 223-237 (2018).
