# Supplementary material for: Functional validation of a human GLUD2 variant in a murine model of Parkinson’s disease
Source: Cell Death Dis. 2020 Oct 22;11(10):897. doi: 10.1038/s41419-020-03043-2 (PMC7582183; doi:10.1038/s41419-020-03043-2)
Supplement: Supplementary file 2 — Supplementary Figure Legends [file 41419_2020_3043_MOESM2_ESM.docx]

**Supplementary Figure Legends**

**Supplementary Figure 1. Effect of *GLUD2* or its mutant on the motor function of untreated mice.** (a-c) Total distance travelled, time spent in the center zone, and number of entries to the center zone in AAV-GFP, AAV-GLUD2, and AAV-GLUD2 T1492G groups. (d) The pole-climbing test was used to examine the bradykinesia of mice. (e) The rotarod test was used to examine the motor coordination of mice. (f) The grasping test was used to examine the grip strength of mice. n = 7 per group. Results are expressed as the mean ± SEM.

**Supplementary Figure 2. Effect of *GLUD2* or its mutant on the striatal TH expression in MPTP-treated mice.** (a) Immunohistochemical staining of TH-positive cells in the striatum of AAV-GFP, MPTP+AAV-GFP, MPTP+AAV-GLUD2, and MPTP+AAV-GLUD2 T1492G groups (scale bars, 1 mm). (b) Expression levels of GFAP, TH and DAT in the striatum of AAV-GFP, MPTP+AAV-GFP, MPTP+AAV-GLUD2, and MPTP+AAV-GLUD2 T1492G groups were determined by Western blotting. (c) Golgi staining was used to stain spines in the striatum of AAV-GFP, MPTP+AAV-GFP, MPTP+AAV-GLUD2, and MPTP+AAV-GLUD2 T1492G groups (scale bars, upper, 50 µm; lower, 10 µm). Western blotting results are from three of the six mice per group and are expressed as the mean ± SEM. ^**^*p* < 0.01, ^*^*p* < 0.05 vs. AAV-GFP Control group. ^##^*p* < 0.01 vs. MPTP+AAV-GFP group. Statistical significance was determined by one-way ANOVAs and Tukey tests for *post-hoc* comparisons.

**Supplementary Figure 3. Effect of *GLUD2* or its mutant on the nigrostriatal TH expression in untreated mice.** (a) Expression levels of GDH2, GDH1, GFAP, TH and DAT in the SN of AAV-GFP, AAV-GLUD2, and AAV-GLUD2 T1492G groups were determined by Western blotting. (b) Expression levels of GFAP, TH and DAT in the striatum of AAV-GFP, AAV-GLUD2, and AAV-GLUD2 T1492G groups were determined by Western blotting. Results are from three mice per group and are expressed as the mean ± SEM. ^**^*p* < 0.01 vs. AAV-GFP Control group. Statistical significance was determined by one-way ANOVAs and Tukey tests for *post-hoc* comparisons.

**Supplementary Figure 4. Effect of *GLUD2* or its mutant on the glutamate uptake in MPTP-treated mice and MPP^+^-treated U251 cells.** (a) The glutamate uptake in the synaptosomes of SN was measured by L-[^3^H]-Glutamic acid uptake assay. (b) The effect of *GLUD2* or its mutant on glutamate uptake in U251 cells was determined by L-[^3^H]-Glutamic acid uptake assay. Results are expressed as the mean ± SEM. ^**^*p* < 0.01 vs. AAV-GFP group or untreated U251 cells. ^##^*p* < 0.01 vs. MPTP+AAV-GFP or MPP^+^ group. ^&^*p* < 0.05 vs. MPTP+GLUD2 or MPP^+^+GLUD2 group. Statistical significance was determined by one-way ANOVAs and Tukey tests for *post-hoc* comparisons.

**Supplementary Figure 5. Metabolomic analysis of *GLUD2* or its mutant for nigral metabolites in MPTP-treated mice.** (a) The relative abundances of representative metabolites in each group are shown. (b) The VIP plots show the significant metabolites among AAV-GFP, MPTP+AAV-GFP, MPTP+AAV-GLUD2, and MPTP+AAV-GLUD2 T1492G groups. The black dots indicate the unchanged metabolites among these four groups, while the green dots indicate the differentially changed metabolites among these four groups.

**Supplementary Figure 6. Effect of *GLUD2* or its mutant on the expression of nigral apoptotic associated proteins in untreated mice.** (a) Quantitative analysis of the numbers of mitochondria in the AAV-GFP, MPTP+AAV-GFP, MPTP+AAV-GLUD2, and MPTP+AAV-GLUD2 T1492G groups. (b) Expression levels of Bax, Bcl-2 and BDNF in the SN of AAV-GFP, AAV-GLUD2, and AAV-GLUD2 T1492G groups that were not treated with MPTP were determined by Western blotting. Results are from three mice per group and are expressed as the mean ± SEM. ^**^*p* < 0.01 vs. AAV-GFP Control group. Statistical significance was determined by one-way ANOVAs and Tukey tests for *post-hoc* comparisons.
