# Supplementary figures and images for: Functional validation of a human GLUD2 variant in a murine model of Parkinson’s disease
Source: Cell Death Dis. 2020 Oct 22;11(10):897. doi: 10.1038/s41419-020-03043-2 (PMC7582183; doi:10.1038/s41419-020-03043-2)

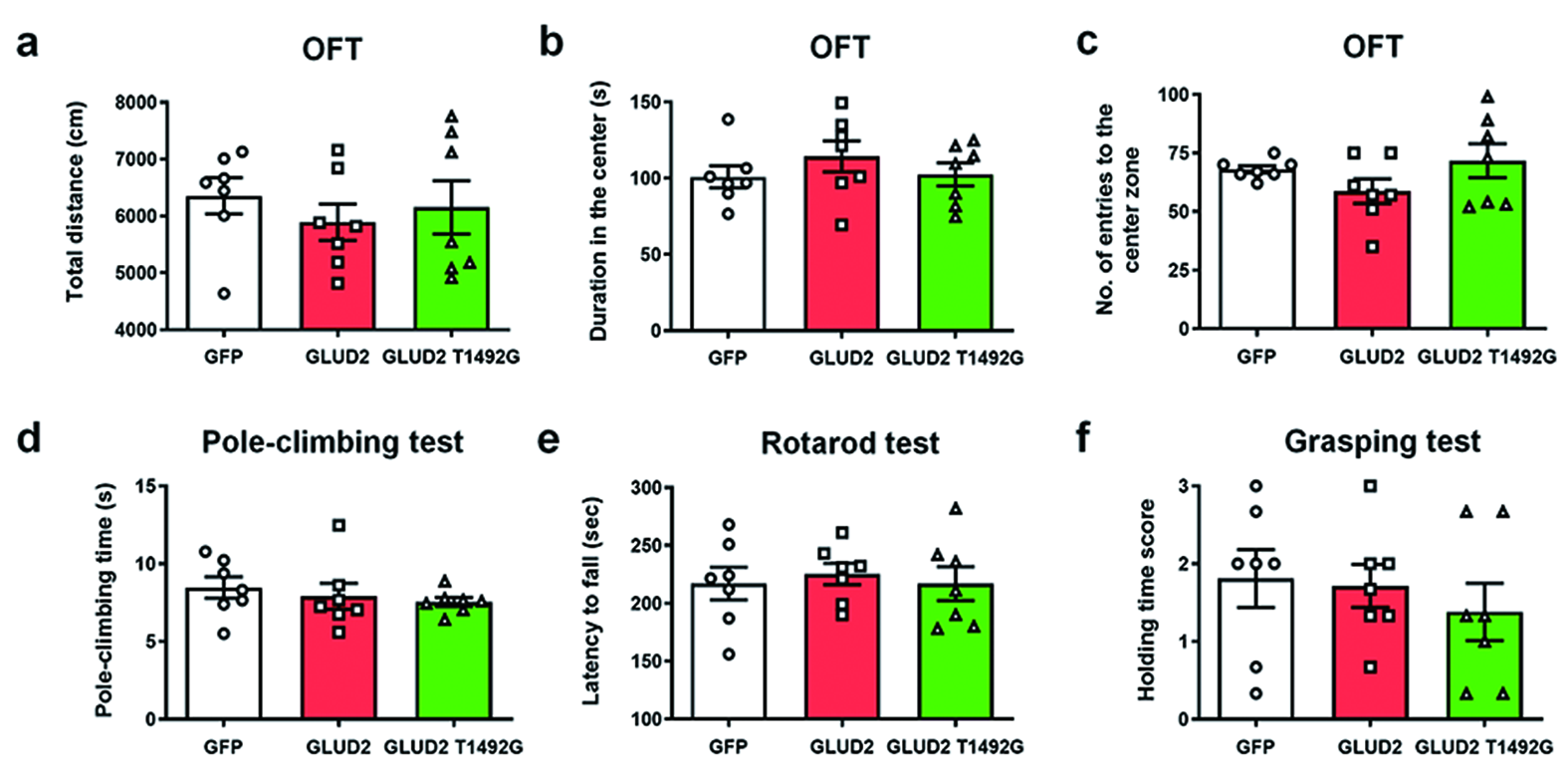

Supplement: Supplementary file 3 — Supplementary Figure 1 [file 41419_2020_3043_MOESM3_ESM.tif]

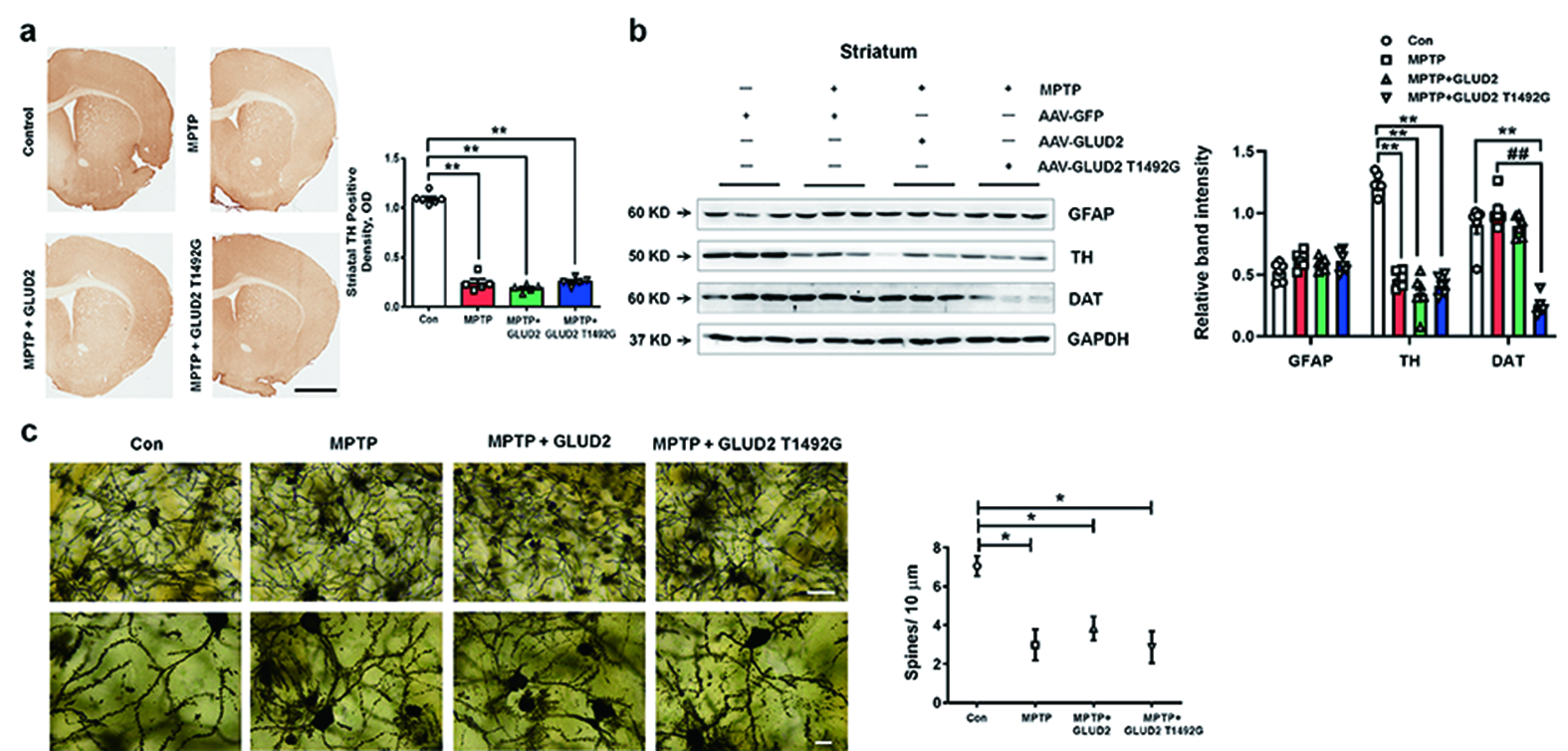

Supplement: Supplementary file 4 — Supplementary Figure 2 [file 41419_2020_3043_MOESM4_ESM.tif]

**a****Substantia nigra**

|   |   |   |
|---|---|---|
| + | — | — |
| — | + | — |
| — | — | + |

AAV-GFP

AAV-GLUD2

AAV-GLUD2 T1492G

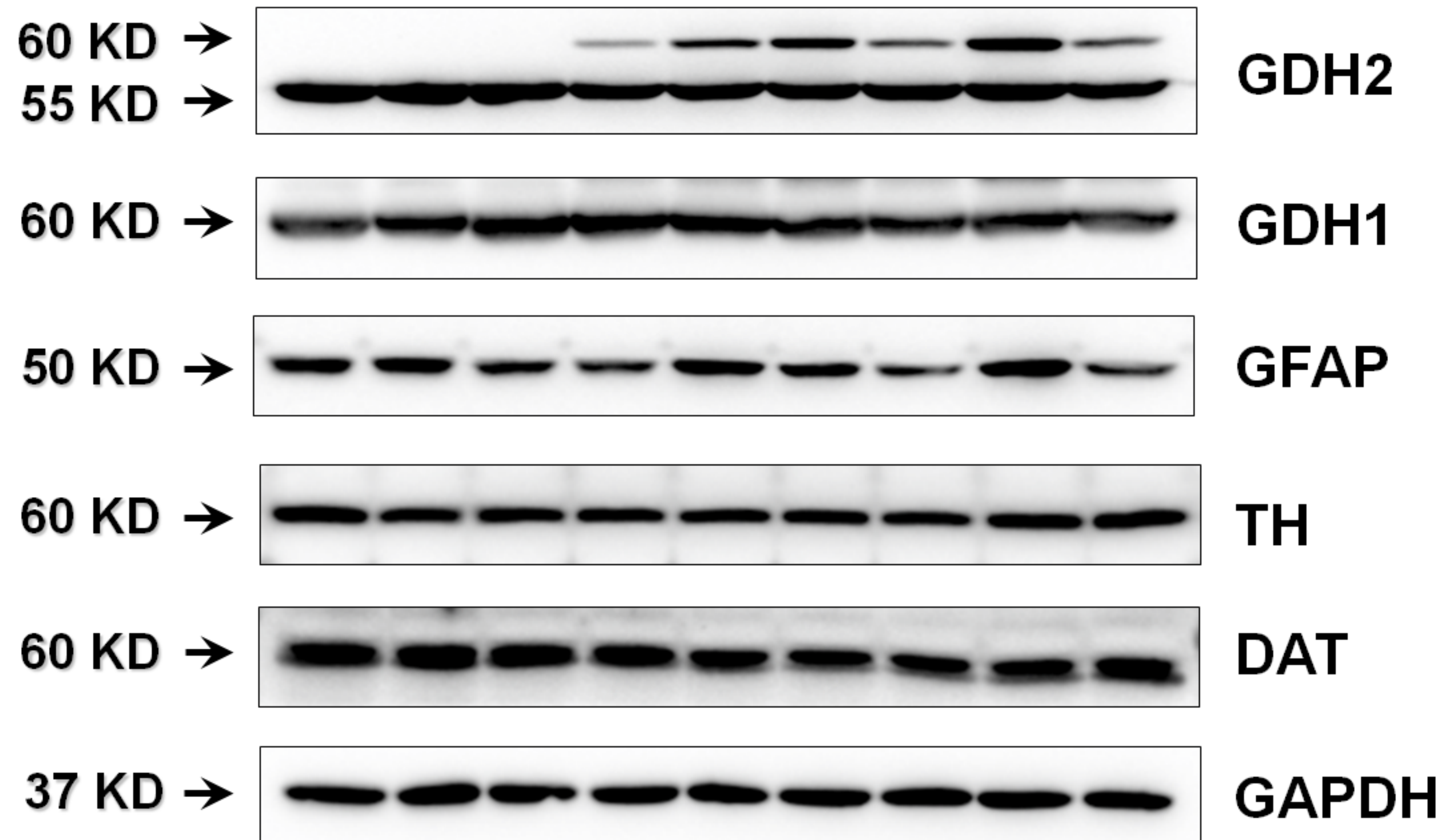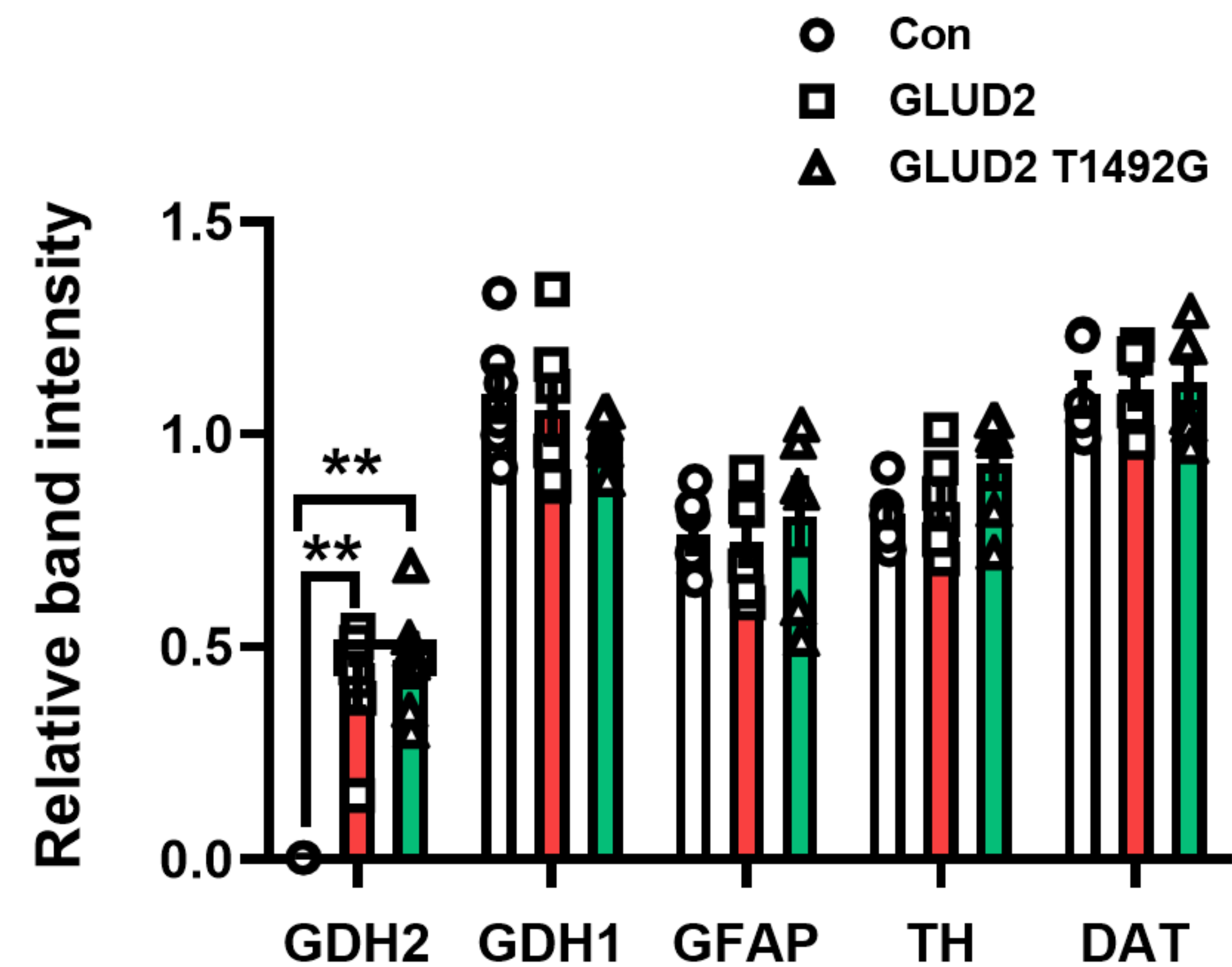**b****Striatum**

|   |   |   |
|---|---|---|
| + | — | — |
| — | + | — |
| — | — | + |

AAV-GFP

AAV-GLUD2

AAV-GLUD2 T1492G

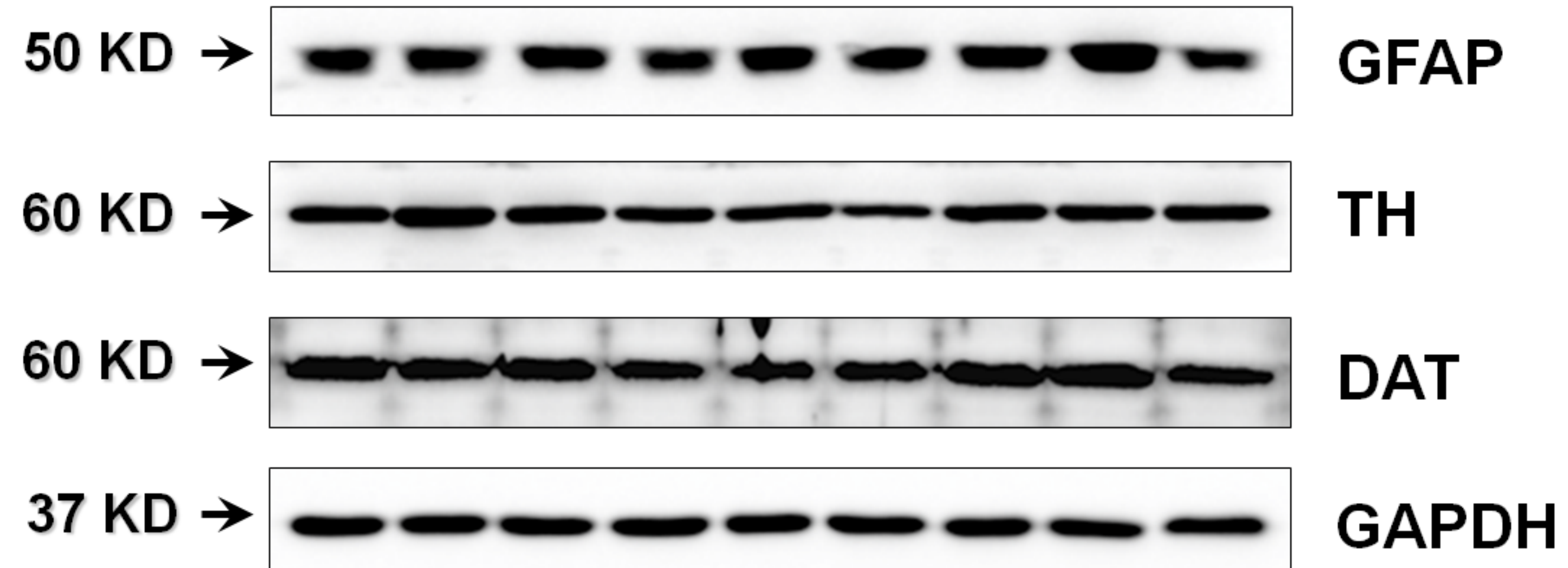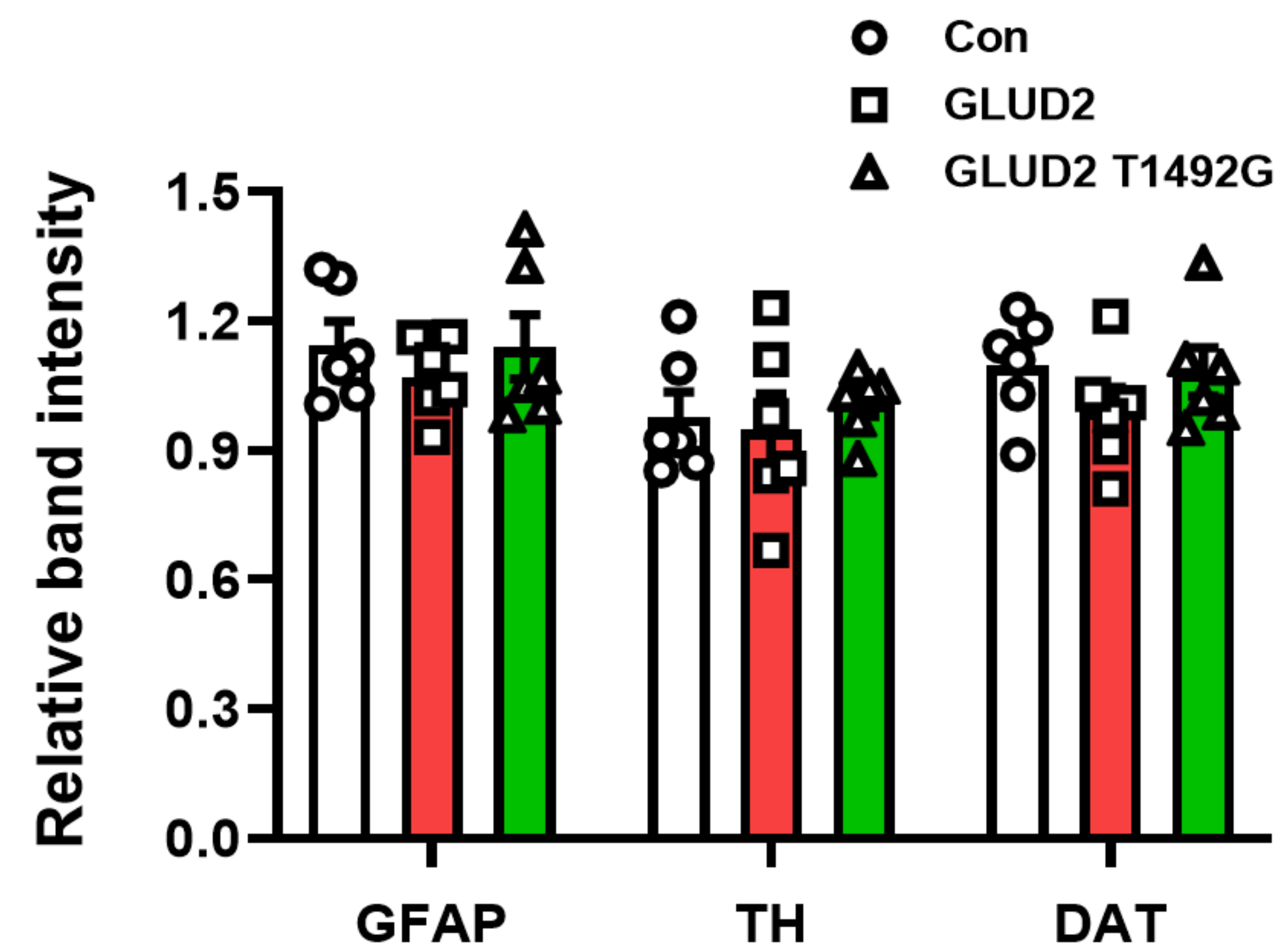

Supplement: Supplementary file 5 — Supplementary Figure 3 [file 41419_2020_3043_MOESM5_ESM.pdf]

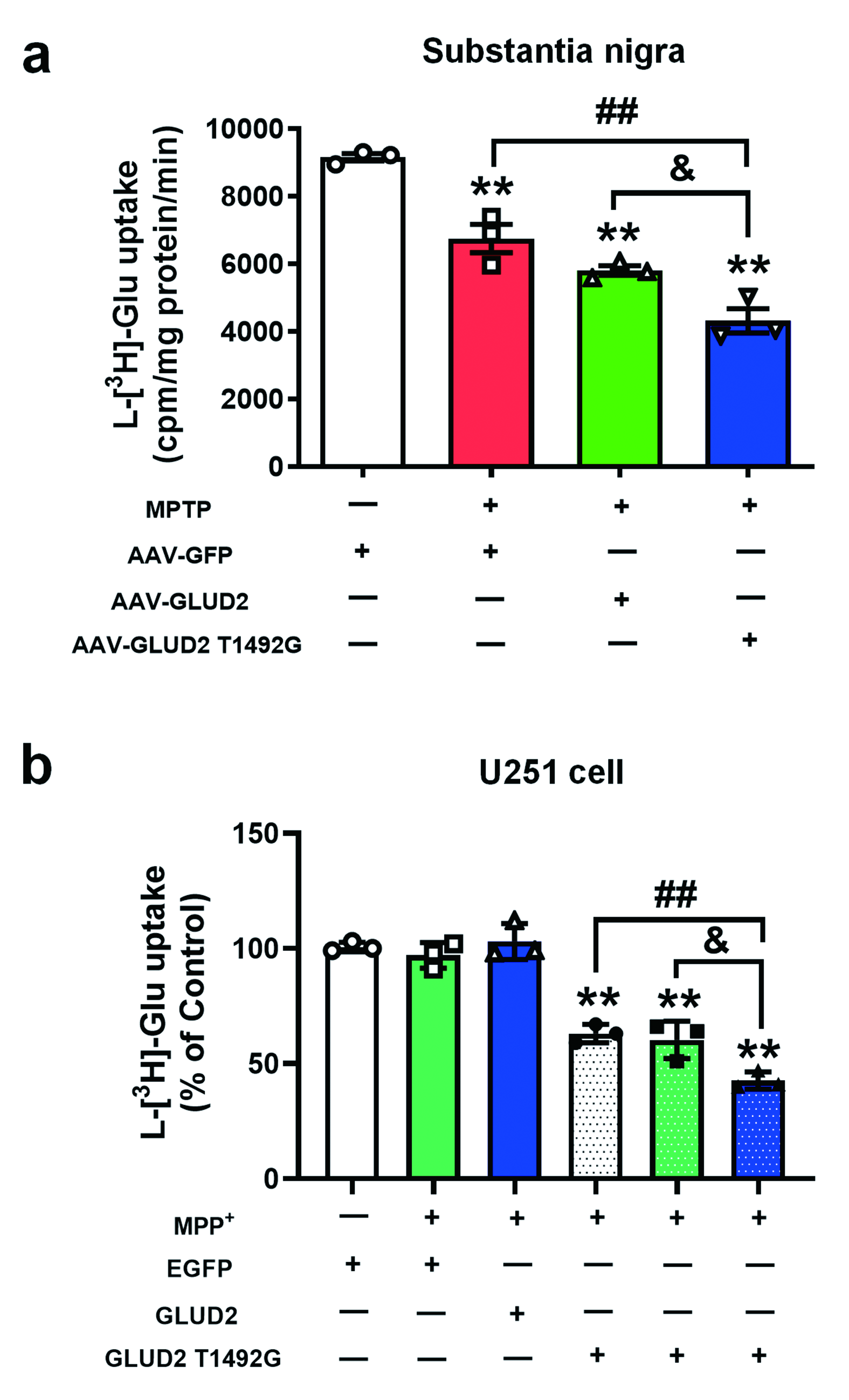

Supplement: Supplementary file 6 — Supplementary Figure 4 [file 41419_2020_3043_MOESM6_ESM.tif]

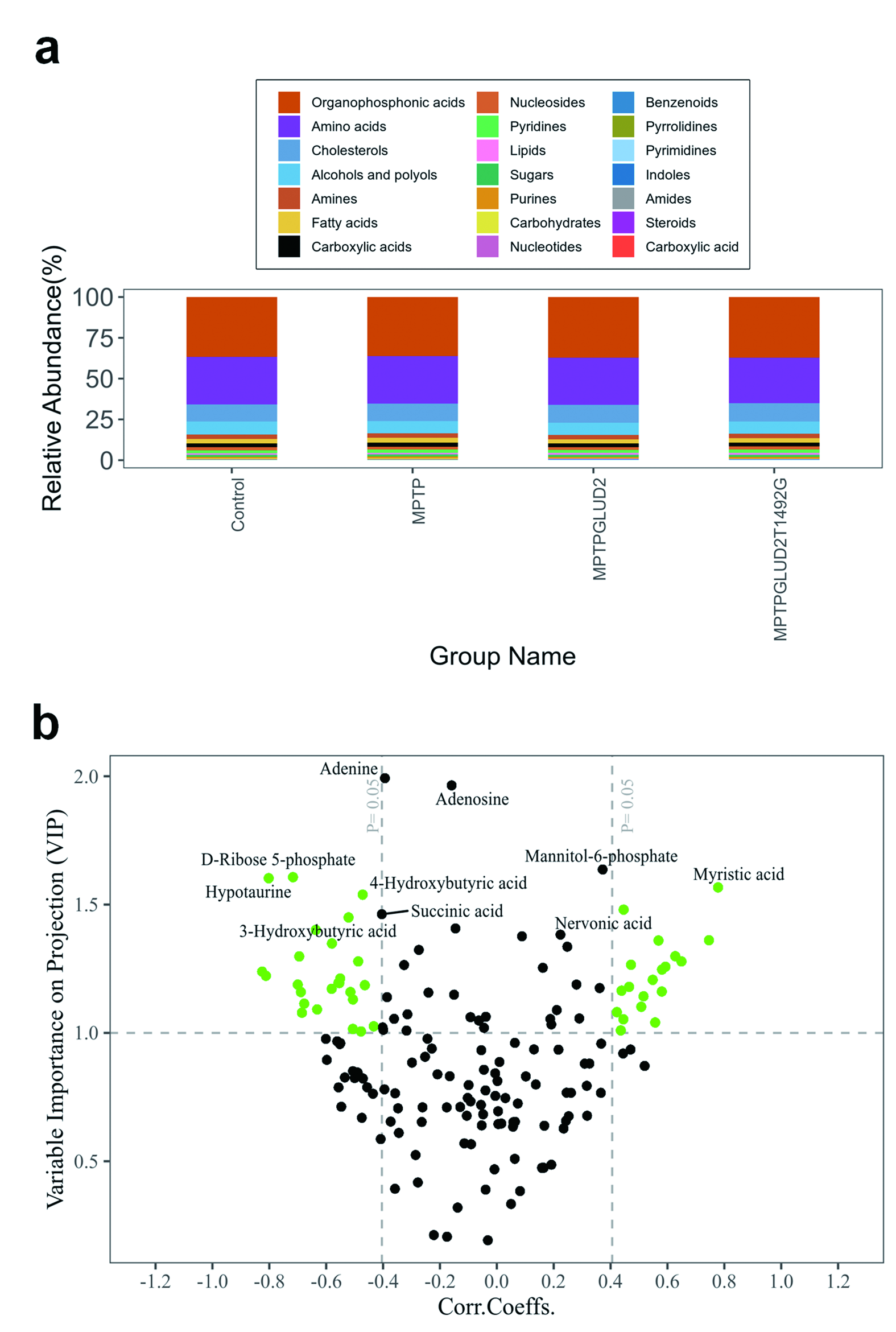

Supplement: Supplementary file 7 — Supplementary Figure 5 [file 41419_2020_3043_MOESM7_ESM.tif]

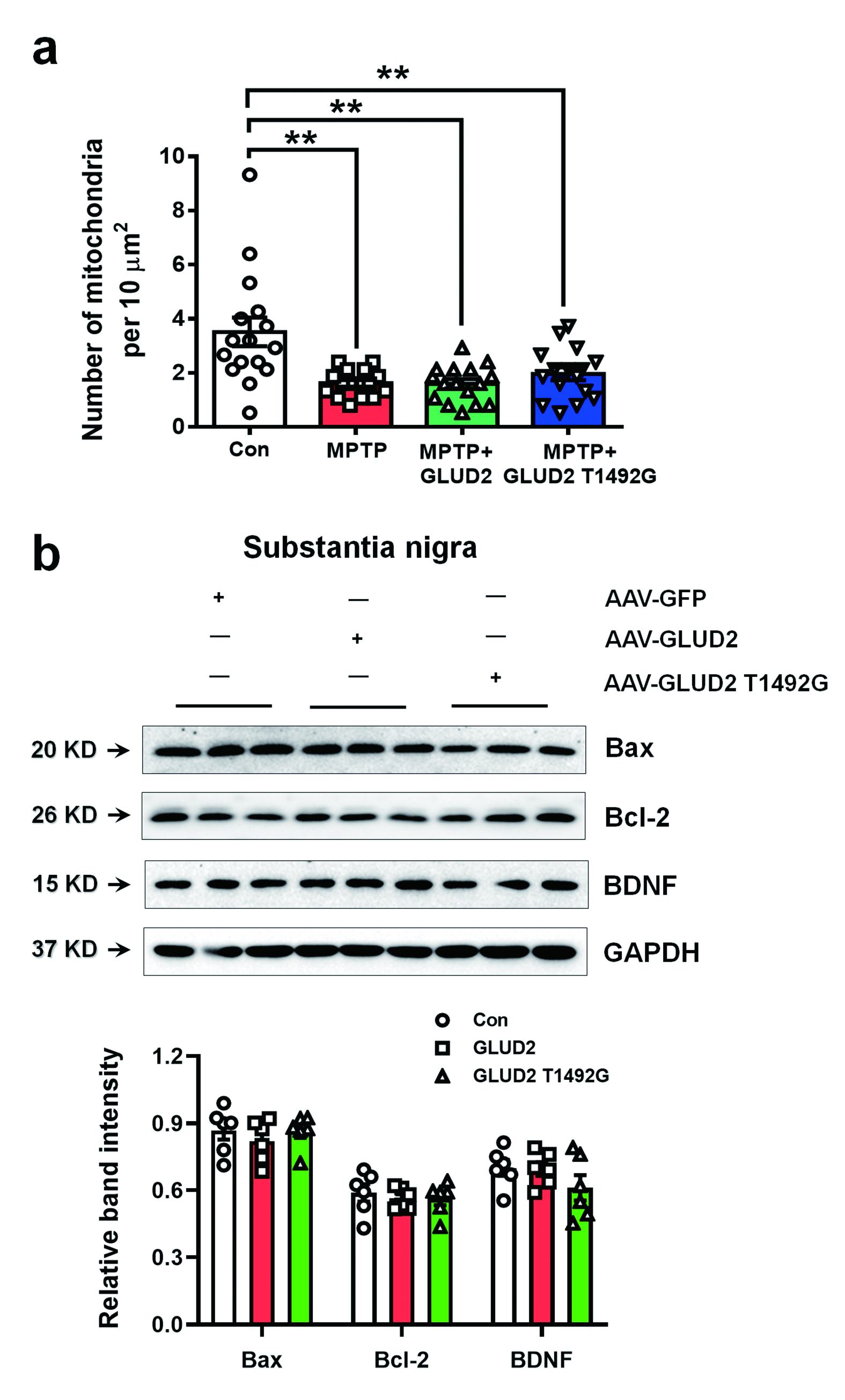

Supplement: Supplementary file 8 — Supplementary Figure 6 [file 41419_2020_3043_MOESM8_ESM.tif]
